# Imprinting of metal receptors into multilayer polyelectrolyte films: fabrication and applications in marine antifouling†
†Electronic supplementary information (ESI) available: FTIR, NMR spectra of synthesized polymers, XPS spectra and AFM images of non-cross linked and cross linked LBL_A_ and LBL_B_ films, UV-Visible absorption spectra of copper complexation with PAH-His, QCM data of LBL_A_ and LBL_B_ films and stability of the films are provided in the electronic supplementary information. See DOI: 10.1039/c4sc02367f



**DOI:** 10.1039/c4sc02367f

**Published:** 2014-09-26

**Authors:** Sreenivasa Reddy Puniredd, Dominik Jańczewski, Dewi Pitrasari Go, Xiaoying Zhu, Shifeng Guo, Serena Lay Ming Teo, Serina Siew Chen Lee, G. Julius Vancso

**Affiliations:** a Institute of Materials Research and Engineering , A*STAR (Agency for Science, Technology and Research) , 3 Research Link , 117602 , Singapore . Email: janczewskid@imre.a-star.edu.sg ; Fax: +65 6872 0785 ; Tel: +65 6874 5443; b Tropical Marine Science Institute , National University of Singapore , 18 Kent Ridge Road , 119227 , Singapore; c Institute of Chemical and Engineering Sciences , A*STAR , 1, Pesek Road , Jurong Island , 627833 , Singapore . Email: g.j.vancso@utwente.nl ; Fax: +31 53 4893823 ; Tel: +31 53 489 2974; d MESA+ Institute for Nanotechnology , Materials Science and Technology of Polymers , University of Twente , P.O. Box 217 , 7500 AE Enschede , The Netherlands

## Abstract

Polymeric films constructed using the layer-by-layer (LbL) fabrication process were employed as a platform for metal ion immobilization and applied as a marine antifouling coating. The novel Cu^2+^ ion imprinting process described is based on the use of metal ion templates and LbL multilayer covalent cross-linking. Custom synthesized, peptide mimicking polycations composed of histidine grafted poly(allylamine) (PAH) to bind metal ions, and methyl ester containing polyanions for convenient cross-linking were used in the fabrication process. Two methods of LbL film formation have been investigated using alternate polyelectrolyte deposition namely non-imprinted LbL_A_, and imprinted LbL_B_. Both LbL films were cross linked at mild temperature to yield covalent bridging of the layers for improved stability in a sea water environment. A comparative study of the non-imprinted LbL_A_ films and imprinted LbL_B_ films for Cu^2+^ ion binding capacity, leaching rate and stability of the films was performed. The results reveal that the imprinted films possess enhanced affinity to retain metal ions due to the preorganization of imidazole bearing histidine receptors. As a result the binding capacity of the films for Cu^2+^ could be improved by seven fold. Antifouling properties of the resulting materials in a marine environment have been demonstrated against the settlement of barnacle larvae, indicating that controlled release of Cu ions was achieved.

## Abstract 2

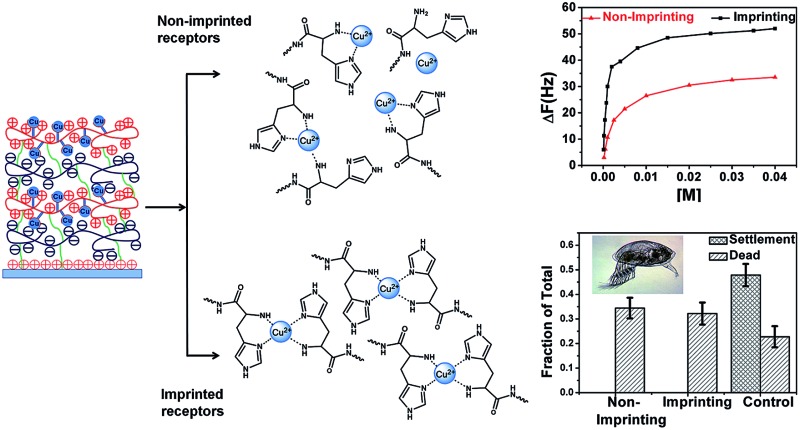
Polymeric films constructed using layer-by-layer fabrication were employed as a platform for metal ion immobilization and applied as a marine antifouling coating.

## Introduction

The ongoing quest for complex, advanced materials systems necessitates the development of various thin polymeric films for applications in a variety of fields including membrane technology,^1–3^ microelectronics,^4^ optical sensing,^5^ and biomedical applications.^6,7^ Particular attention is directed toward the management of the molecular payloads within the film *e.g.* selective loading, and controlled delivery or multiplexed release.^8,9^ The layer-by-layer (LbL) electrostatic fabrication process utilizing alternate deposition of positively and negatively charged polyions on a substrate^10^ is a simple yet versatile method for the preparation of thin polyelectrolyte multilayer coatings.^11–14^ Polymeric films with enhanced affinity toward metal ions are needed for applications in ion capture and separation,^1–3,15^ catalysis,^16,17^ protein binding, metal affinity chromatography^18^ and antimicrobial materials.^19^ The selective adsorption and separation of metal ions from aqueous waste has also received increasing consideration in environmental protection in recent years.^20^ For example, numerous adsorbents have been developed for the removal of copper ions from industrial wastewater and in other applications.^21,22^ However, these adsorbents still suffer from drawbacks such as limited selectivity and low reusability. Molecular-imprinting techniques (MIPs) have become acclaimed in many fields, such as chemosensor fabrication, molecular separation, and catalysis and drug delivery.^23–27^ Ion imprinted polymers can be considered as a sub category within MIPs^22,27^ and refer to materials with improved affinity toward metals by the pre-organization of metal receptors within polymeric matrix in the presence of metal ion templates. This process is typically achieved by the reduction of receptor mobility through various ways such as bulk polymerization, precipitation polymerization, and suspension polymerization. Since the receptor sites are embedded deeply into the polymeric matrix, these imprinting techniques often produce materials exhibiting high selectivity but low rebinding capacity and poor site accessibility to target ions.^28^ In order to conquer these drawbacks, thin film surface-ion-imprinted polymers were proposed as a promising solution. Films obtained by surface ion-imprinted techniques exhibit good characteristics, such as complete removal of templates and fast adsorption kinetics.^29–33^ Small molecules and ions have been loaded into non-cross-linked LbL fabricated thin electrostatic film systems using both the traditional solution, the LbL method,^16,34^ as well as spray deposition approaches.^35^ The films obtained show not only a high affinity to metals but also possess reversible loading–unloading capability.^36^ Enhanced selectivity of such films toward the ions loaded is attributed to the reduced mobility of polyions upon deposition in the electrostatic multilayer.^37^ This affinity however can potentially be further increased by the reduction of receptor mobility, for example through covalent cross-linking of the LbL layers. The ion imprinting through covalent cross-linking of multilayer systems in the presence of metal templates has not been presented until now. Studies, which employed cross-linking of LbL systems in the presence of ions have focused only on moderately coordinating the polyethylenimine/polyacrylic acid (PEI/PAA) couple. Enhanced affinity of the films toward specific metals upon cross-linking has not been demonstrated, nor discussed, in the corresponding reports.^38,39^ Finally, the direct imprinting of metals into a polyelectrolyte film by the immobilization of metal templated receptors was demonstrated only for a single PEI layer (non LbL) using an epichlorohydrin crosslinker.^40,41^ However a single polymer layer is inferior compared to LbL coatings in terms of substrate coverage and the number of receptors available. Reported systems used also rather poorly coordinating PEI receptors.

Protein complexes with metal ions play an important role in various biological processes.^42^ A common structural feature of such protein-metal assemblies^43^ are peptide motifs typically arranged in a special sequence of residues to ensure the strong interactions between the metal ions and donor atoms from the peptide backbones or functional side groups.^29,44^ These motifs are frequently bearing residues such as histidine (His), since nitrogen of the imidazole moiety is known for its high affinity to metals.^45,46^ It is used as a metal coordinating component of polymeric systems and it can be grafted on various polymeric backbones mimicking peptide architecture.^47–49^ Hence in this work we shall describe a system in which this specific coordinating capacity of histidine is utilized.

Marine fouling has been a serious problem for maritime technologies, from ship hull protection to off-shore stationary structures and high value-added underwater sensing and communications.^50,51^ Marine fouling has been controlled traditionally through the use of antifouling paints with toxic constituents or biocides.^52^ Before its ban tributyltin (TBT) was the active agent in antifouling paints and used extensively in the maritime sector for over thirty years.^53^ As an alternative, copper based coatings are ten–twelve folds less harmful than those containing TBT,^54,55^ however still remain problematic and are criticized for excessive metal pollution. Copper is used as an antifouling agent with a leaching rate in the order of 10-15 μg cm^–2^ per day. Hence a surface of one square meter can be kept clean of biological growth by annual leaching of approximately 30 grams of copper.^56^ Those levels are suspected to be toxic to aquatic organisms, through the accumulation in filter feeders, such as mussels, and damage larval stages of aquatic invertebrates and fish species.^57^ Designing and fabricating new, effective and environmentally friendly coating systems as alternatives to TBT-based antifouling paints poses an important challenge for the scientific community. The ideal antifouling coating with embedded metal ions would prevent marine growth as well as maintain a long performance life whilst fulfilling the expectations of the increasingly strict environmental regulations.

In this work we investigate the concept of improving of thin LbL polymeric film affinity to Cu^2+^ ions by reducing the mobility of metal receptors in the presence of guest molecules. This concept is typically referred to as metal ion imprinting.^22^ Utilizing the properties of the polymer grafted imidazole bearing histidine receptor, we synthesized a peptide mimicking material with high affinity to copper. Two alternative approaches for the fabrication of metal attracting films were compared. In the first imprinting approach, thin LbL films composed of custom synthesized histidine grafted polyallyamine were cross-linked in the presence of Cu^2+^ ions to immobilize receptors in a conformation suitable for the metal ion coordination. In the second non-imprinting approach, the films were cross-linked without the presence of ions to provide a reference material. The structure and properties of both films are extensively characterized. Finally, anti-fouling applications of the copper ion selective LbL films are demonstrated.

## Experimental

### Materials

Poly(isobutylene-*alt*-maleic anhydride) (PIAMA, Mw: 6 kDa), poly(allylamine hydrochloride) (PAH, Mw: 58 kDa), l-histidine methyl ester dihydrochloride, 3-aminopropyltrimethoxysilane (APTMS), N,N-diisopropylethylamine (DIPEA), copper(ii) nitrate trihydrate (Cu(NO_3_)_2_ × 3H_2_O), sodium chloride, sea salt and sodium hydroxide (all from Sigma Aldrich), N,N-dimethylformamide (DMF), dimethylsulfoxide (DMSO), toluene, methanol, acetone and isopropanol (all from Tedia) were used directly as received without further purification. Dialysis membrane tubing (MWCO: 3.5 kD) was received from Fisher Scientific. Silicon wafers (Latech Scientific Supply Pte. Ltd) were 0.6 mm thick, with one side polished and with a natural silicon dioxide layer. QSX 303 Silicon dioxide 50 nm quartz crystal microbalance (QCM) chips were obtained from Analytical Technologies Pte Ltd. Deionized (DI) water with 18 MΩ cm^–1^ resistivity was obtained from a Millipore Nanopure system.

### Synthesis

#### Synthesis of PIAMA-Ester (PIAMA-Me)

**Scheme 1:**
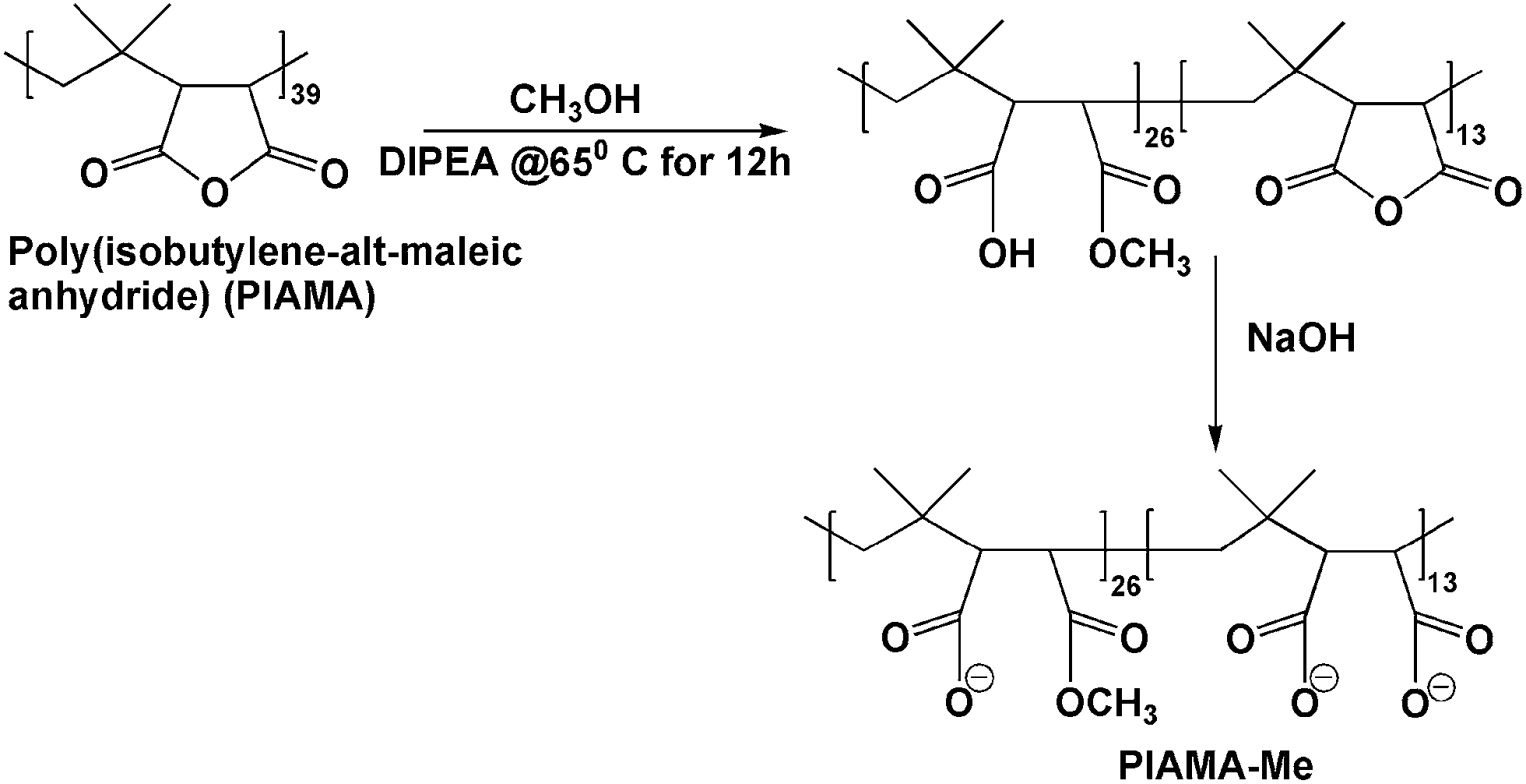
The synthesis of PIAMA-Me.

PIAMA-Me synthesis was achieved following a modification of the method described previously (Scheme 1).^58^ To the solution of poly(isobutylene-*alt*-maleic anhydride) (1.0 g, 6.5 mmol) in 300 mL of methanol, DIPEA (1.2 mL, 6.9 mmol) was added and the mixture was stirred for 16 h at 50°C. After the evaporation of methanol and DIPEA, the material was suspended in water using a small excess of NaOH with respect to the carboxylic groups in the polymer backbone. The polymer solution was dialyzed against 0.01 M NaOH and subsequently against pure water for a few days. The purified polymer aqueous solution was concentrated by rotary evaporator and finally freeze dried to yield the solid polymer. To properly identify the composition of the polymers obtained, NMR spectra were compared with the NMR results of poly (isobutylene-*alt*-maleic anhydride) opened by a treatment with a stoichiometric amount of NaOH to carboxyl groups. NMR calculated Mn: 8 kDa. ^1^H NMR integrated for a single repeating unit: (D_2_O) *δ*_H_: 0.5-1.36 ppm (6H, m), 3.67 ppm (2H, s). IR: 1860 cm^–1^, 1780 cm^–1^, 1730 cm^–1^, 1580 cm^–1^.

#### Synthesis of PAH-Histidine (PAH-His)

**Scheme 2:**
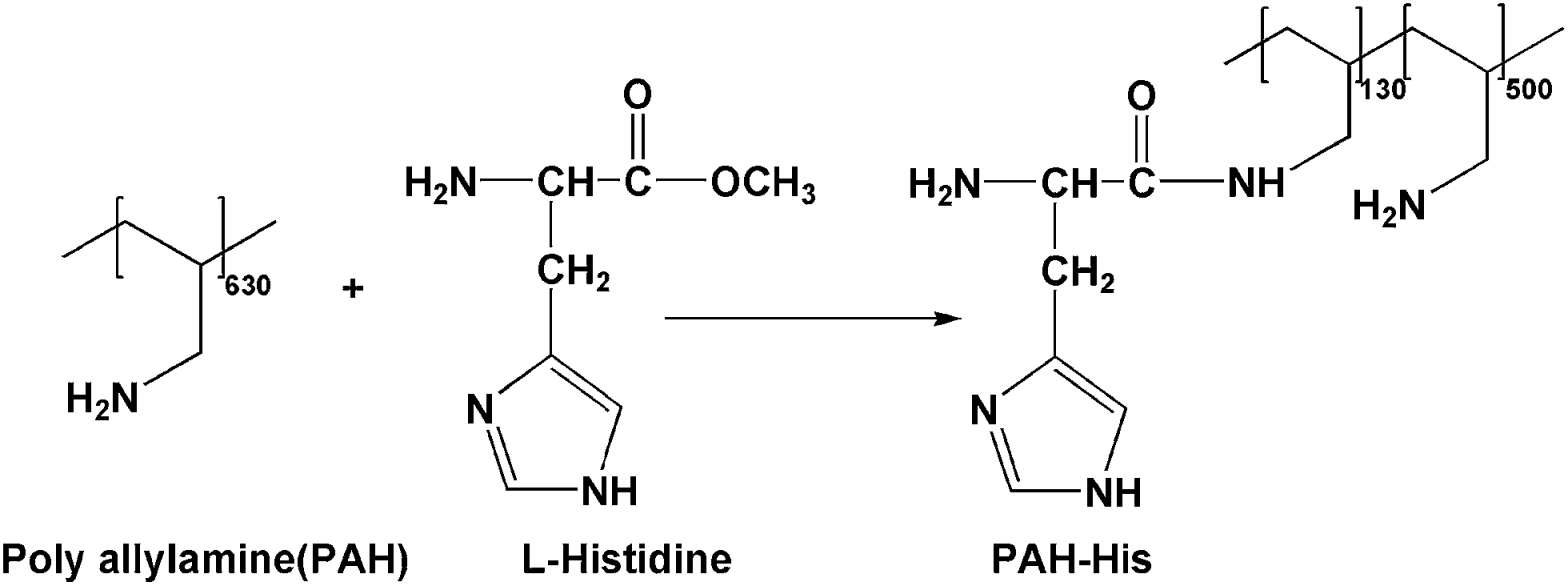
The synthesis of PAH-His.

To a solution of l-histidine methyl ester (2.63 g, 11 mmol) in 20 mL of DI water, 1 g of PAH (7.4 mmol of the repeating units) was added in small portions (Scheme 2). The solution was stirred for 1 h at room temperature and small portions of 5M NaOH were added until the solution pH reached 10. The solution was freeze dried for 72 h. 2 mL of DMSO was added to the freeze dried polymer-histidine mixture and kept under vacuum at 65°C for 72 h. After evaporation of DMSO, the remaining polymer was dissolved in water and dialyzed against diluted HCl for 12 h and against pure water for several days. The polymer solution was concentrated by rotary evaporator and freeze dried to yield a white solid powder (1.7 g, yield 81.7%). NMR calculated Mn: 80 kDa. ^1^H NMR integrated for a single repeating unit: (D_2_O) *δ*_H_: 0.5-1.76 ppm (3H, m), 4.05 ppm (0.2H, s), 7.10 ppm (0.22H, s) and 7.85 ppm (0.21H, s). IR: 620 cm^–1^, 1260 cm^–1^, 1750 cm^–1^.

### Binding constant evaluation from the solution

Optical absorption spectra were recorded using a UV-VIS spectrometer to investigate the complexation of copper ions with PAH-His and to establish equilibrium metal-binding constants for PAH-His in solution. In all experiments, water was used as a reference. A copper ion titration was performed by adding aliquots of a Cu(NO_3_)_2_ stock solution (15 mM) to 6 mM of PAH-His solution in water. All calculations and model fittings were carried out using MS Excel software.

### Surface modification

Silicon substrates were cut into 2 × 2 cm^2^ slides by a DISCO dicing machine (DAD 321), cleaned with acetone and isopropanol in ultrasonic bath for 10 min, rinsed with DI water and finally dried with a stream of nitrogen. Subsequently, silicon slides were treated by oxygen plasma (200 W) for 2 min in a triple P plasma processor (Duratek, Taiwan). This cleaning procedure created a surface rich in hydroxyl groups at the oxide surface to facilitate the subsequent silanization process. The hydroxyl-covered substrates were rinsed with methanol–toluene mixture and toluene, and then immersed in a 3 mM APTMS solution in toluene for 3 h. Subsequently, they were rinsed copiously with toluene, sonicated for 10 min, rinsed again successively with toluene, methanol and water and blown dry with nitrogen and dried under vacuum at 100°C for 5 h.

### Polymeric film fabrication

PIAMA-Me, PAH-His and 1 mM of Cu(NO_3_)_2_ × 3H_2_O complexed with PAH-His (referred to in this work as PAH-His(Cu)) stock solutions (1 mg mL^–1^) were prepared by dissolving the corresponding solids in 0.5 M NaCl. The synthesized PAH-His and PAH-His(Cu) solutions had the pH values of pH = 6.5 and pH = 5.5, respectively. The cleaned silicon wafers were treated with APTMS to impart positive charges onto the surface. Subsequently, the substrates were immersed into the PIAMA-Me solution for 10 min, rinsed with DI water for 2 min and dried with a flow of nitrogen gas. In the second step, the substrates were immersed into the PAH-His solution for 10 min, followed by 2 min of rinsing with DI water and blown dry with nitrogen. This cycle was repeated until the desired number of 14 bi-layers was reached. PAH-His was always deposited as the outermost layer. Such fabricated films were cross-linked by heating the silicon substrate at 80°C for 12 h under vacuum.^58,59^ The described process of LbL assembly of PIAMA-Me and PAH-His polymers is referred to as non-imprinted LbL_A_ in this work (Fig. 2 and 3).

**Fig. 1:**
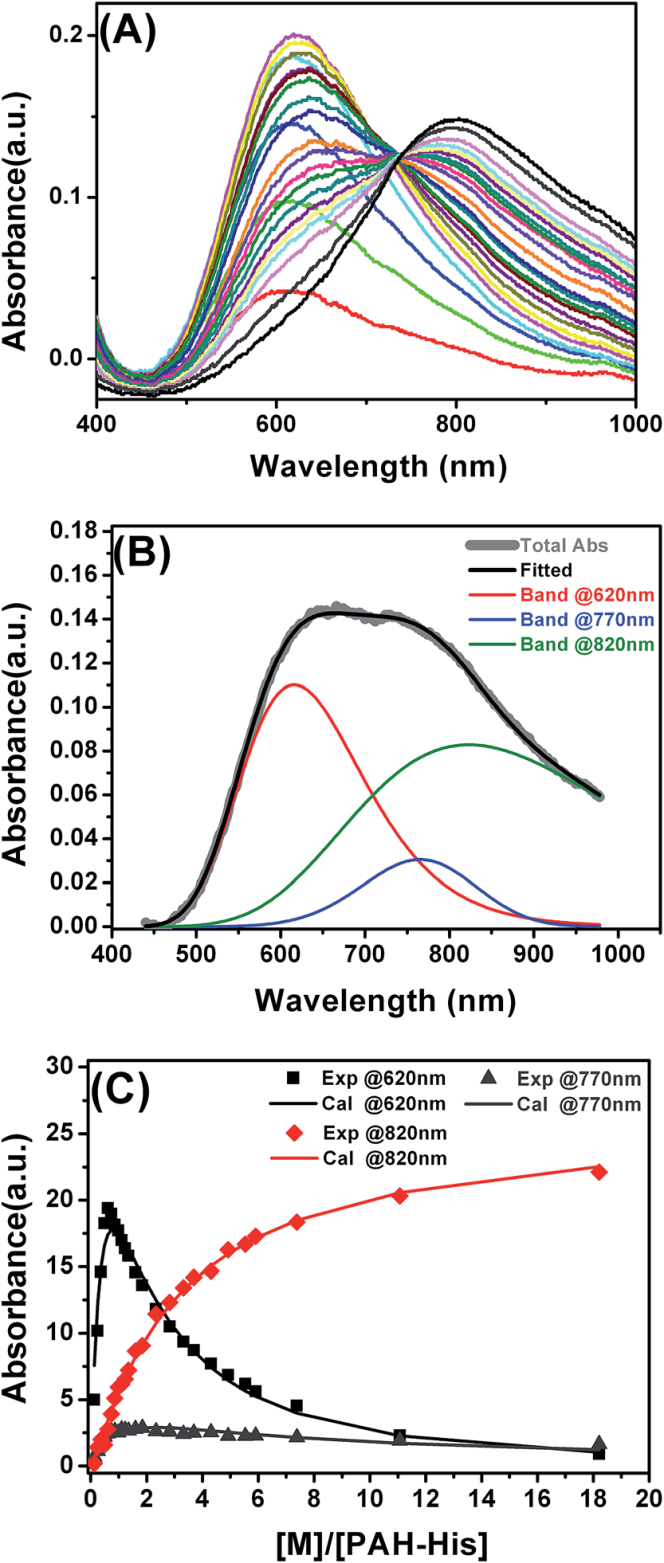
(A) UV-VIS absorption spectra of PAH-His coordinated with Cu. (B) Example of absorption spectra fitted with three different signals for 2.3 mL of PAH-His (6 mM) added to the 2 mL of Cu(NO_3_)_2_ (15 mM). (C) Integration of the signal intensity at 620 nm, 770 nm and 820 nm, fitting data to the binding model.

Cross-linked LbL films with Cu^2+^ ions preloaded at the fabrication stage were obtained by alternating immersions of the silicon slides prepared as described above, but using polymer solutions PIAMA-Me and PAH-His(Cu). 18 bi-layers (PIAMA-Me and PAH-His(Cu)) were assembled in this way using PAH-His(Cu) as the outermost layer. The process of LbL assembly of PIAMA-Me and PAH-His(Cu) polymers is referred to as imprinted LbL_B_ in this work (Fig. 1).

### LbL films-loading and releasing of copper ions

**Fig. 2:**
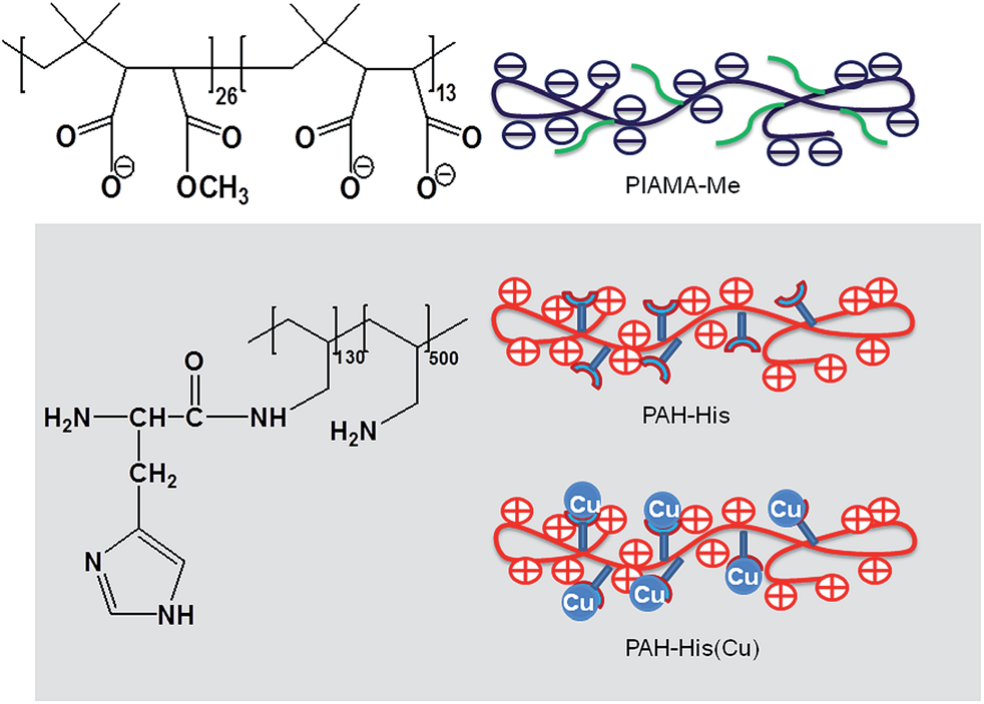
Schematic illustration of PIAMA-Me, PAH-His and PAH-His(Cu).

**Fig. 3:**
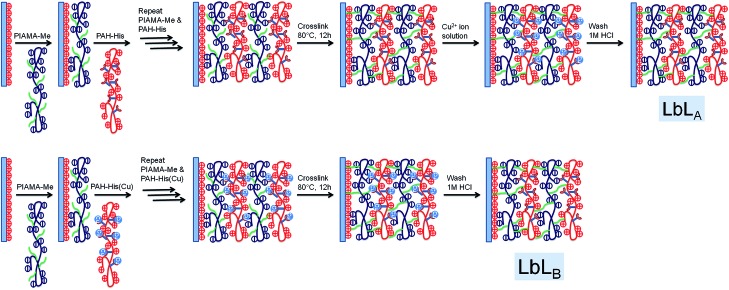
LbL film formation by different processing methods, (non-imprinted LbL_A_ refers to assembly of PIAMA-Me and PAH-His, and imprinted LbL_B_ refers to assembly of PIAMA-Me and PAH-His(Cu)). Cross linking of the films as employed followed by loading and releasing of copper within the LbL films. Schematic representation of the polymer PIAMA-Me, PAH-His and PAH-His(Cu) are shown in Fig. 2.

The preparation process of the Cu^2+^ imprinted LbL_B_ and non-imprinted LbL_A_ films on silicon substrates are illustrated in Fig. 2 and 3. The loading and releasing of the copper ions was achieved by the following procedure. Imprinted LbL_B_ films were first washed using 1 M HCl for 5 min to remove the Cu^2+^ ions as the Cu^2+^ were present during the LbL process, and copious amounts of DI water, followed by drying with a stream of nitrogen. Non-imprinted LbL_A_ and imprinted LbL_B_ films were immersed in 15 mM solution of Cu(NO_3_)_2_ to absorb the Cu^2+^ ions for 1h to ensure that equilibrium was reached and the films were washed with copious amounts of water and dried with nitrogen. In the next step, Cu^2+^ ion-loaded non-imprinted LbL_A_ and imprinted LbL_B_ films were rinsed with 1 M HCl for 5 min to extract the Cu^2+^ ions followed by washing with DI water and drying with nitrogen. The above procedure is repeated several times to load and release the copper from the films. The non-imprinted LbL_A_ and imprinted LbL_B_ films with Cu^2+^ ions were investigated using XPS to ensure that there were no copper ions left on the surface after washing with HCl.

### LbL assembly and metal loading monitored by QCM

*In situ* quartz crystal microbalance (QCM) measurements were conducted using a Qsense E4 multichannel instrument equipped with silicon dioxide-coated crystals. The surface of silicon dioxide chips were modified with APTMS similar to the silicon wafers as described above. For *in situ* QCM measurements, the as-prepared silane modified QCM chips were fixed in the chamber of the QCM instrument and the crystals were sealed with a silicon rubber O-ring. Then the APTMS films were stabilized in a flow of water followed by 0.5 M NaCl until the frequency reached equilibrium at the flow rate of 250 μL min^–1^. PIAMA-Me polymer solutions of (1 mg mL^–1^ in 0.5 M NaCl) were passed through the chamber at a rate of 250 μL min^–1^ until the frequency reached equilibrium followed by the injection of 0.5 M NaCl through the chamber at the same rate until the frequency became stable enough to remove any loosely bound molecules. Afterwards PAH-His polymer solutions (1 mg mL^–1^ in 0.5 M NaCl) were pumped at the same flow rate followed by a NaCl washing step (non-imprinted LbL_A_). Similarly, in imprinted LbL_B_ films, alternate injections of PIAMA-Me and PAH-His(Cu) polymer solutions in 0.5 M NaCl were delivered at a flow rate of 250 μL min^–1^ until the frequency became stable, followed by 0.5 M NaCl salt injection after every polymer layer was deposited. The procedure was repeated until the required number of bi-layers was reached. Finally, the layers were rinsed with DI water at a flow rate of 250 μL min^–1^ to remove any possible excess of salt. Each layer deposition was carried out for 30 min to 1 h to achieve saturated adsorption. The frequency change of the quartz crystals was monitored throughout the adsorption process. The crystals were removed from the QCM instrument and dried with nitrogen. Cross linking of the LbL_A_ and LbL_B_ films grown on QCM chips were performed at 80°C for 12 h under vacuum.

Cross-linked non-imprinted LbL_A_ films built on QCM chips were immersed in 15 mM of Cu(NO_3_)_2_ solutions for 1 h and washed with 1M HCl, similar to the silicon wafers. Cross-linked films prepared by the imprinted LbL_B_ method on QCM were washed with 1 M of HCl for 5 min and dried. For copper loading experiments, non-imprinted LbL_A_ and imprinted LbL_B_ films on QCM chips, after washing with HCl as described above, were loaded into the chamber of the QCM instrument. Subsequently, the LbL films were stabilized in a flow of water at a rate of 250 μL min^–1^ until the frequency reached equilibrium. Then the sequence of different concentrations of Cu(NO_3_)_2_ stock solutions (0.1, 0.2, 0.4, 1, 2, 4, 8, 15, 25, 35 and 45 mM) were injected. For each concentration 250 μL of the Cu(NO_3_)_2_ solutions were injected and the frequency was allowed to reach equilibrium followed by injection of 1 mL of DI water through the chamber at the rate of 250 μL min^–1^. The injection of water was intended to remove any loosely bound Cu^2+^. This procedure was repeated for all concentrations. The frequency change of the quartz crystal was monitored throughout the loading of copper ions. Finally, the crystals were removed from the QCM instrument and dried with nitrogen. Cu^2+^ ions loaded from non-imprinted LbL_A_ and imprinted LbL_B_ films on QCM chips were washed with 1 M of HCl for 5 min and copious amounts of DI water to remove copper ions, followed by dry blowing with nitrogen. The above procedure was repeated several times to load and unload the Cu^2+^ ions using the QCM process on non-imprinted LbL_A_ and imprinted LbL_B_ films. The mass deposited on the crystal (Δ*m*) was calculated using the Sauerbrey equation.^60^

1

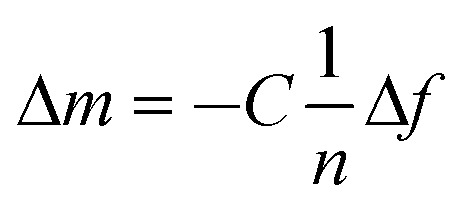




For a 5, 15, 25, 35, 45 MHz quartz crystal, *n* = 1, 3, 5, 7; where C = 17.7 ng s^–1^ cm^–2^, *n* = overtone number and Δ*f* = frequency change.

### Stability

Stability and the leaching rate of copper from the cross linked non-imprinted LbL_A_ and imprinted LbL_B_ films were evaluated by immersing them in artificial sea water solutions. The silicon wafers with 7 bi-layers films from non-imprinted LbL_A_ and 10 bi-layers from imprinted LbL_B_ were immersed in artificial seawater (prepared by dissolving 38.5 g L^–1^ sea salt in DI water), for up to 90 days. Samples were removed and rinsed by DI water after 1, 7, 15, 45 and 90 days of immersion. The surfaces were characterized by XPS after drying with nitrogen.

### Larval culture

*Amphibalanus amphitrite* barnacle larvae were spawned from adults collected from the Kranji mangrove, Singapore. The nauplii larvae were fed with an algal mixture 1 : 1 v/v of *Tetraselmis suecica* (CSIRO Strain number CS-187) and *Chaetoceros muelleri* (CSIRO Strain number CS-176) at a density of ∼5 × 10^5^ mL^–1^, and reared at 27 °C in 2.7% salinity, 0.2 μm filtered seawater (FSW). Under these conditions, the nauplii metamorphosed into cyprids in 5 days. The cyprids were aged for 2 days at 4–6 °C prior to use in settlement assays.

### Barnacle settlement and toxicity assay

The assay method as described by Wang *et al.* ^61^ was adapted in this work. Positive controls consisted of plasma-cleaned silicon wafers. A 200 μL drop of filtered seawater (FSW) containing 15–25 barnacle cyprids were added to each wafer kept in individual Petri dishes. In addition, 100 μL of FSW containing 15–25 stage 2 barnacle nauplii was introduced to each droplet giving a final volume of 300 μL. The control, as well as the non-imprinted LbL_A_ and imprinted LbL_B_ films with copper loaded samples were incubated in the dark at 26–28 °C and the results were scored after 24 hours. For the assessment of barnacle settlement, juvenile barnacles and metamorphosing cyprids that had attached firmly to the substrate were counted as ‘settled’. The number of dead nauplii and cyprids were also enumerated for an assessment of toxicity.

## Results and discussion

To investigate the concept of molecular imprinting of metal receptors in the LbL electrostatic thin film matrix and to employ this material in the fabrication of antifouling polymeric coatings, two custom synthesized polyelectrolytes were developed. The polymer PIAMA-Me was constructed by the opening of polyanhydride units with two nucleophilic agents, namely methanol and hydroxide. The resulting polyanion is bearing negatively charged carboxylic groups and ester functions for covalent cross-linking with amine (Fig. 2 and 3).^58^ The results of FTIR analysis confirm the partial conversion of the anhydride polymer (PIAMA) to ester (PIAMA-Me) through the methanolysis reaction as shown in Scheme 1. The double peak (1860 cm^–1^ and 1780 cm^–1^) belonging to the C=O of the anhydride polymer (PIAMA) disappears and a new peak at 1730 cm^–1^, attributed to the C=O of the ester bond, becomes visible. In addition, a peak at 1580 cm^–1^ can be associated to a newly formed COO^–^ (Fig. S1A in the ESI†).^62^ The methanolysis reaction of PIAMA was further verified by the comparison of the ^1^H NMR spectra of PIAMA-Me and purified polyanhydride as shown in Fig. S2 (ESI†). The presence of a peak at 3.66 ppm in the polymer PIAMA-Me spectrum, belonging to the protons of methyl ester, confirms successful conversion. The methyl ester peak (3.66 ppm) integrated area, and the dimethyl group protons of the main chain (peaks between 0.5–1.36 ppm) allow us to estimate the degree of substitution with the methyl esters groups to obtain the value of 70% (Scheme 1).

The polycation (PAH-His) was synthesized by the grafting of l-histidine side groups on the polyallylamine backbone. Following this approach, peptide mimicking polymer bearing histidine moieties for metal ion complexation and primary amine groups used in the LbL assembly was synthesized (Scheme 2). The successful polymer synthesis was confirmed by the appearance of peaks at 620 cm^–1^ and 1260 cm^–1^, which are assigned to OCN bending and amide C–N stretching and N–H bending frequencies respectively. The ester peak visible at 1750 cm^–1^ in pure histidine, and it’s absence in the PAH-His spectrum (see ESI Fig. S1B†) as well as the presence of the peaks at 1260 cm^–1^ (amide C–N stretching) and at 1570 cm^–1^ (amide carbonyl) in the PAH-His polymer spectrum, confirm successful chemical transformation.^63^ The PAH-His structure and histidine substitution degree were examined and quantitatively evaluated by ^1^H-NMR as shown in Fig. S3 (ESI†). The reaction completion is confirmed by the disappearance of the methyl ester peak at 3.67 ppm and the presence of new peaks at 4.05, 7.10 and 7.85 ppm, which can be associated to the methine and imidazole protons in the His moiety, respectively. The backbone substitution degree is determined to 20% owing to the clearly visible signal of imidazole protons (7 and 8 ppm) and the aliphatic backbone proton peaks between 1 and 2 ppm. This allowed us to estimate the indices of the repeating units, describing the composition of polymer PAH-His, to 130 and 500 for PAH-substituted and non-substituted groups respectively (Scheme 2).

### Complexation of copper ions in aqueous solutions

UV-visible absorption spectroscopy experiments were employed to establish the mechanism of interactions between the synthesized polymer (PAH-His) and Cu^2+^ ions in solution. The titration of PAH-His with Cu(NO_3_)_2_ in water followed by the observation of absorption changes allowed us to propose a binding model for these molecules. On the basis of previous reports,^44,47^ we employed the working hypothesis that two histidine ligands bind to a single copper ion. To simplify the model, repeating histidine units of the polymer were treated as individual; independently binding molecules and polyallylamine interactions with ions were omitted. This is justified since primary amine interactions with Cu^2+^ are reported to be much weaker compared to imidazole complexation.^18,36,64^ As shown in Fig. 1A, the addition of copper nitrite to the PAH-His solutions results in the appearance of a new peak at 620 nm. The peak intensity increases continuously with the addition of copper and reaches the absorption maxima approximately for a 1 : 2 metal to ligand ratio. After the saturation, the intensity of the signal at 620 nm decreases with a clear shoulder increase at longer wavelengths. Upon further metal addition a new signal near 820 nm appears which can be assigned to free Cu^2+^ ions.^44,65^ All of the UV absorption spectra were fitted by three peaks representing the concentration of three different species present in the solution using the asymmetric modification of the Gaussian function.^66,67^ The peak at 620 nm is attributed to the formation of a 1 : 2 copper complex with PAH-His, the peak around 770 nm corresponds to the formation of a 1 : 1 copper complex with PAH-His and a peak at 820 nm is linked to free copper ions (Fig. 1B and S4†).^44^ Examples of the fitting procedure are provided in the ESI.†

The integrated absorption values for the three signals are provided in Fig. 1C. The changes observed in the signal intensity variations were subsequently used to establish the binding constants of this system. Titration curves were fitted by assuming that 1 : 1 and 1 : 2 equilibriums are present^68^ as described by the equations 2–5.

2 M + PAH-His ⇔ M(PAH-His)

3

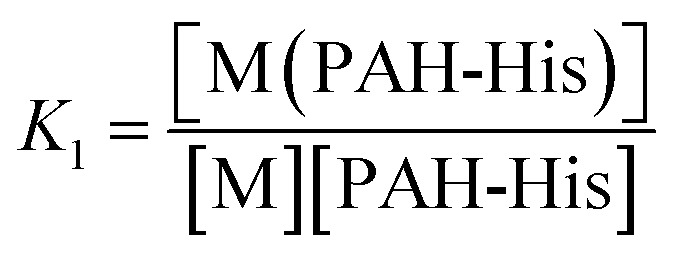

4 M(PAH-His) + PAH-His ⇔ M(PAH-His)_2_

5

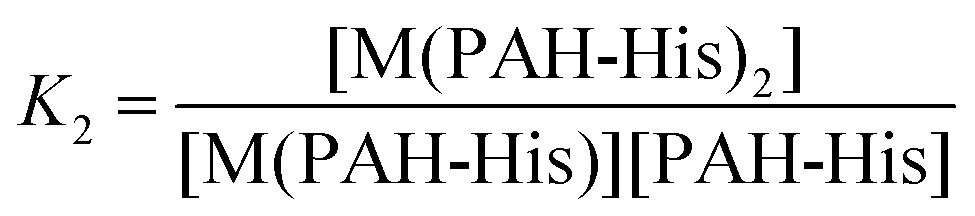




The absorbance signals presented in Fig. 1C clearly support the co-existence of the 1 : 1 and 1 : 2 stoichiometry of the Cu^2+^ and PAH-His complex. The fitting of the data provides binding constant values of *K*_1_ = 8.43 × 10^3^ M^–1^ (log *K*_1_ = 3.93) and *K*_2_ = 2.63 × 10^4^ M^–1^ (log *K*_2_ = 4.42). Based on the above observations it appears that the 1 : 2 metal–PAH-His coordination dominates the 1 : 1 interactions. The results are consistent with a large stability constant observed for other histidine grafted polymers.^44,47^

### LbL film fabrication

Synthesized polymers were used for the fabrication of thin film structures using alternating deposition of polyelectrolytes in a typical electrostatic LbL protocol,^10–13^ followed by the interlayer film cross-linking performed through amide bond formation.^58,59^ Two different designs were compared to investigate the effect of metal imprinting on the affinity of the polymeric matrix to bind Cu^2+^ ions. The imprinting of ion receptors was achieved by the immobilization of histidine moieties around preorganized metal atoms in the covalent, interlayer cross-linking process (Fig. 3).

**Fig. 4:**
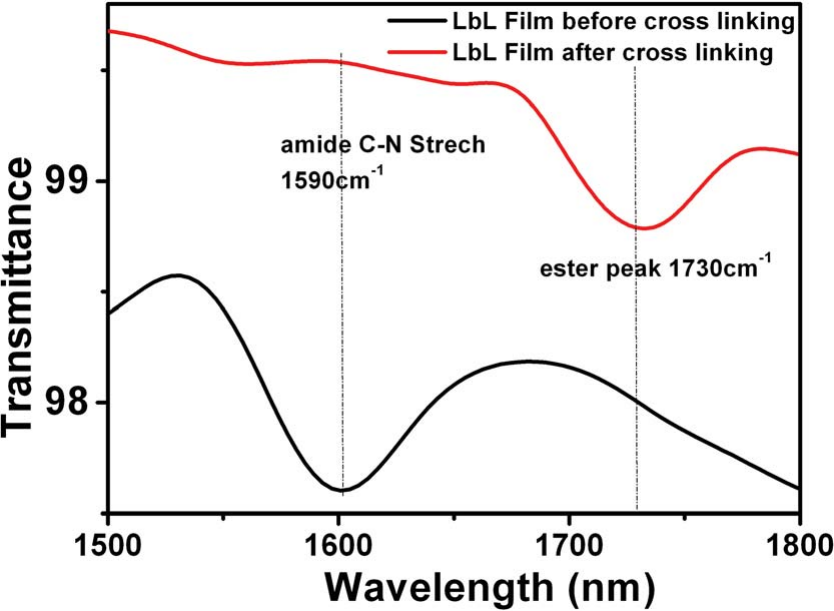
FTIR spectra of non-imprinted LbL_A_ film before and after cross linking.

The two methods used essentially differ in the cross-linking step. Non-imprinted LbL_A_ films are cross-linked without the metal template present and as such, the mobility of the receptors is reduced, maintaining random arrangement not influenced by the presence of copper. In contrast, for the imprinted LBL_B_ system, the film is loaded with Cu^2+^ ions at the stage of fabrication, so the subsequent cross-linking process is intended to “freeze” histidine receptors in the given conformation around the guest molecules (Fig. 3). By using these approaches, two types of structures with similar chemical composition but different arrangement of metal receptors can be obtained. Film cross-linking evidence was acquired using FTIR and XPS methods. The disappearance of the ester peak (1730 cm^–1^) in the non-imprinted LbL_A_ film and the evolution of a new peak at 1590 cm^–1^ (amide C–N stretch) indicate amide bond formation (Fig. 4) and successful covalent attachment between the layers.^62^

**Fig. 5:**
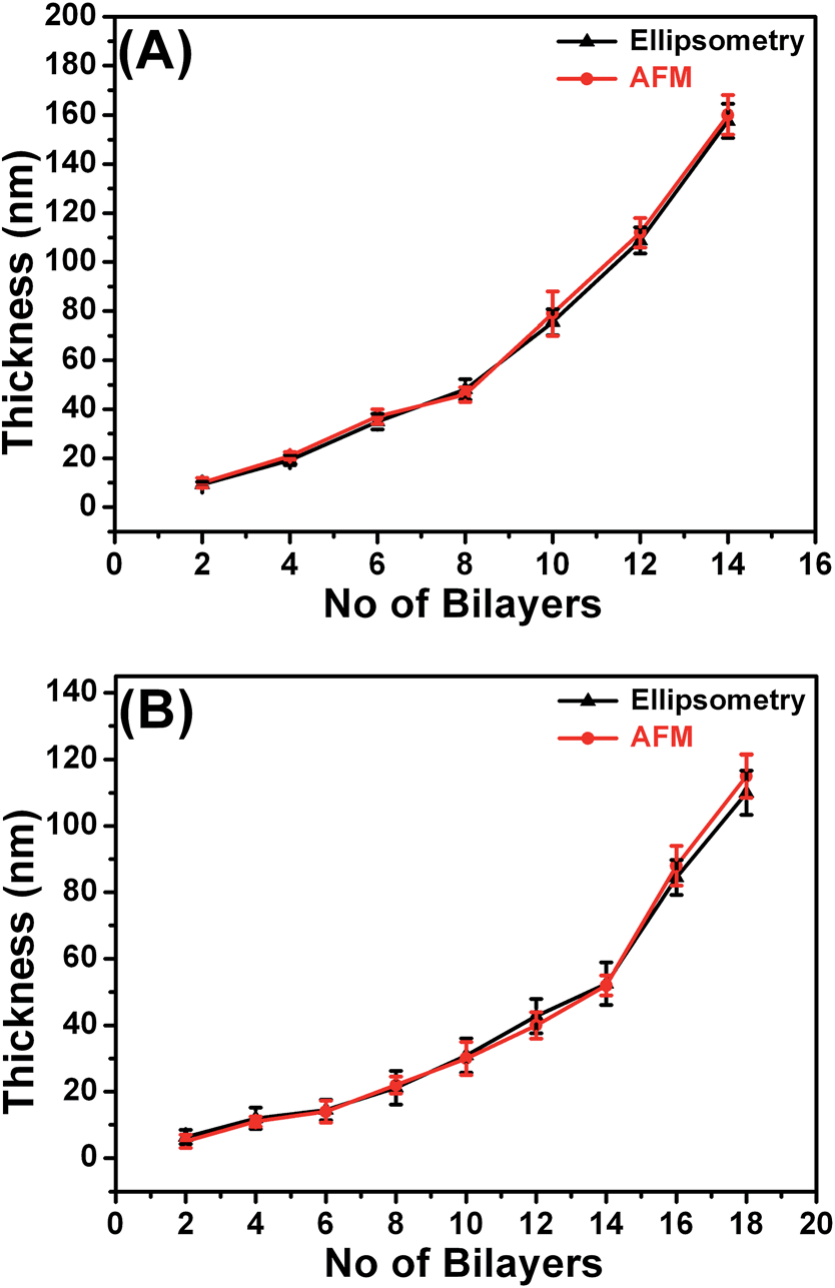
Thickness data of non-imprinted LbL_A_ (A) and imprinted LbL_B_ (B) films from ellipsometry and the AFM scratch test.

The XPS spectra (Fig. S5 in the ESI†) further confirmed the cross-linking of the LbL films in addition to the FTIR analysis. Fig. S5A and B† are the N1s core-level spectra for non-cross linked and cross-linked LbL_A_ films, respectively, which are fitted into primary amine, amide, imide and protonated amine components with binding energy (B.E.) values of 398.9 eV, 399.9 eV, 400.8 and 401.8 eV.^69^ In the spectra of cross-linked non-imprinted LbL_A_ films, the peak component for the free and protonated amines are small. The increase of the amide peak intensity demonstrates that the free amine is reacting with the ester. A similar trend is also observed for the imprinted LbL_B_ films (Fig. S5C and D†). The thickness of the cross linked LbL films were measured by ellipsometry and by the AFM scratch method as shown in Fig. 5. For the non-imprinted LbL_A_, the thickness growth of the films is linear up to 8 bi-layers and exponential afterwards, following the trends reported in the literature (Fig. 5A).^70^ Similarly, for the imprinted LbL_B_ we observed linear growth up to 14 bi-layers and exponential increase afterwards. The thickness of the non-imprinted LbL_A_ and imprinted LbL_B_ films were also measured independently using the AFM scratch method. The obtained thickness values correlate well with the ellipsometry measurements as shown in Fig. 5A and B. To achieve a good comparison among the structures studied, films of similar thicknesses and comparable masses of the polymers deposited were used. This corresponds to 7 bi-layers and 10 bi-layers for non-imprinted LbL_A_ and imprinted LBL_B_, respectively.

The morphology of the LbL films was studied using AFM (Fig. S6 in the ESI†). The AFM images in Fig. S6† show clear variations of the film surface depending on the fabrication method applied. Fig. S6A† shows cluster/coil-like features on the non-cross linked LbL_A_ film, and no significant changes in the morphology upon application of the cross-linking procedure (Fig. S6B†). Rather different morphology is observed for LbL_B_ films. The LbL_B_ film is fairly smooth (Fig. S6D†) when compared to LbL_A_ (Fig. S6B†). The surface roughness *Rq* (the quadratic mean value of roughness, obtained over a 5 × 5 μm^2^ surface area) for an LbL_A_ film without cross-linking exhibited a value of *Rq* = 16 ± 0.5 nm (Fig. S6A†) compared to *Rq* = 13 ± 0.5 nm (Fig. S6B†) for the cross-linked film. Similarly, for the imprinted LbL_B_ films, the roughness values of the non-cross linked film (*Rq* = 10 ± 1 nm from Fig. S6C†) decreased to 8 ± 1 nm (Fig. S6D†) after cross-linking. The results are consistent with previous reports showing that the surface roughness values are reduced with an increased cross-linking density.^58,70^

### LbL growth and metal affinity assessed by QCM

**Fig. 6:**
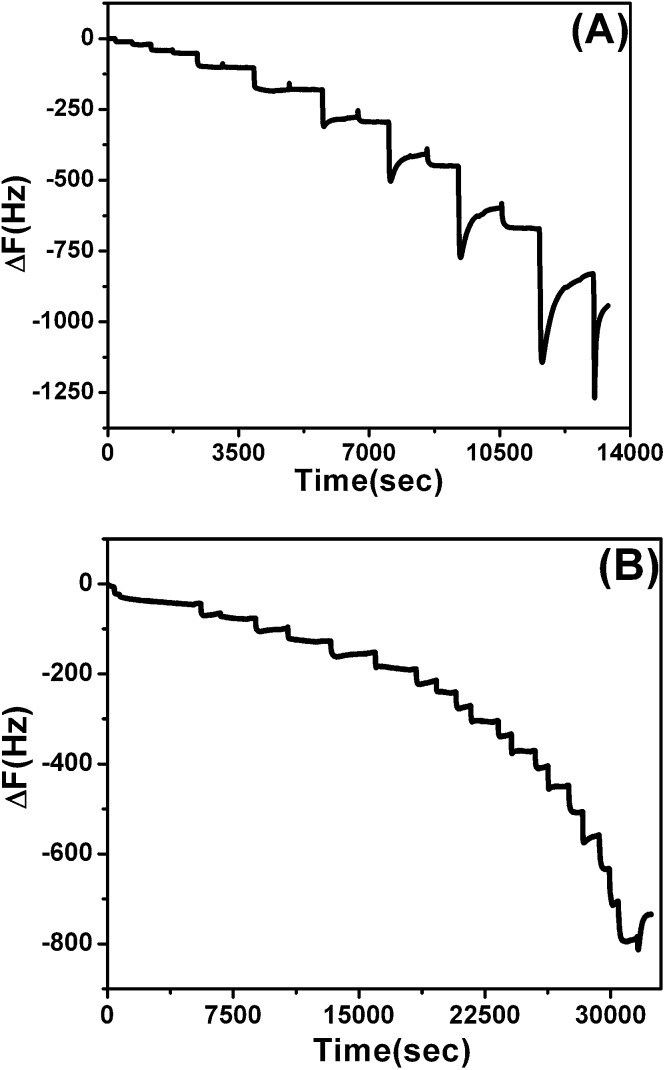
Frequency changes monitored by QCM-D for (A) non-imprinted LbL_A_ (7 bi-layers) and (B) imprinted LbL_B_ (10 bi-layers) films.

To compare LbL_A_ and LbL_B_ film affinity to ions by establishing binding constants for each film and finally to answer the question if the proposed molecular imprinting method allows us to enhance the metal binding capacity of LbL structures, QCM experiments were performed. The buildup of the macromolecules during fabrication of LbL structures on the APTES modified chips, was monitored following frequency changes of QCM signals (Fig. 6). The alternating addition of polycation and polyanion leads to a stepwise rise in the mass for the deposited layers in both non-imprinted and imprinted films (Fig. 6), demonstrating successful layer by layer assembly. The observed increase in the mass of LbL_A_ (Fig. 6A) is higher than LbL_B_ (Fig. 6B). This is most likely related to the pH of the polymer solution. This parameter is lowered by the presence of Cu salt in the PAH-His(Cu), and is responsible for thinner layers of LbL_B_.^71^

Successful LbL deposition is known to be facilitated by the overcompensation of charges, which in turn promotes the binding of the subsequent polyelectrolytes.^10^ Earlier studies suggest that for electrostatically driven LbL deposition both the substrate and the polyelectrolyte species must be sufficiently charged. If the charge is insufficient, the adsorbed layers may be partially removed upon adsorption on the next polyelectrolyte layer.^72^ Similar behavior is observed here when the LbL films were assembled by LBL_A_ with PIAMA-Me and PAH-His polymers. It is also suggested that the reason for such behavior is the formation of free polyelectrolyte complexes in the solution, which are entropically favored in this case compared to a multilayer on the surface.^73,74^ Similar complexes may occur when PAH-His is interacting with PIAMA-Me.

Thickness values found from the QCM data for the cross-linked 7 bi-layer non-imprinted LbL_A_ and the 10 bi-layer imprinted LbL_B_ films are 35 ± 5 nm. The total mass of the PAH-His layers in the non-imprinted LbL_A_ and imprinted LbL_B_ films are 15.9, and 9.3, μg cm^–2^, respectively.

**Fig. 7:**
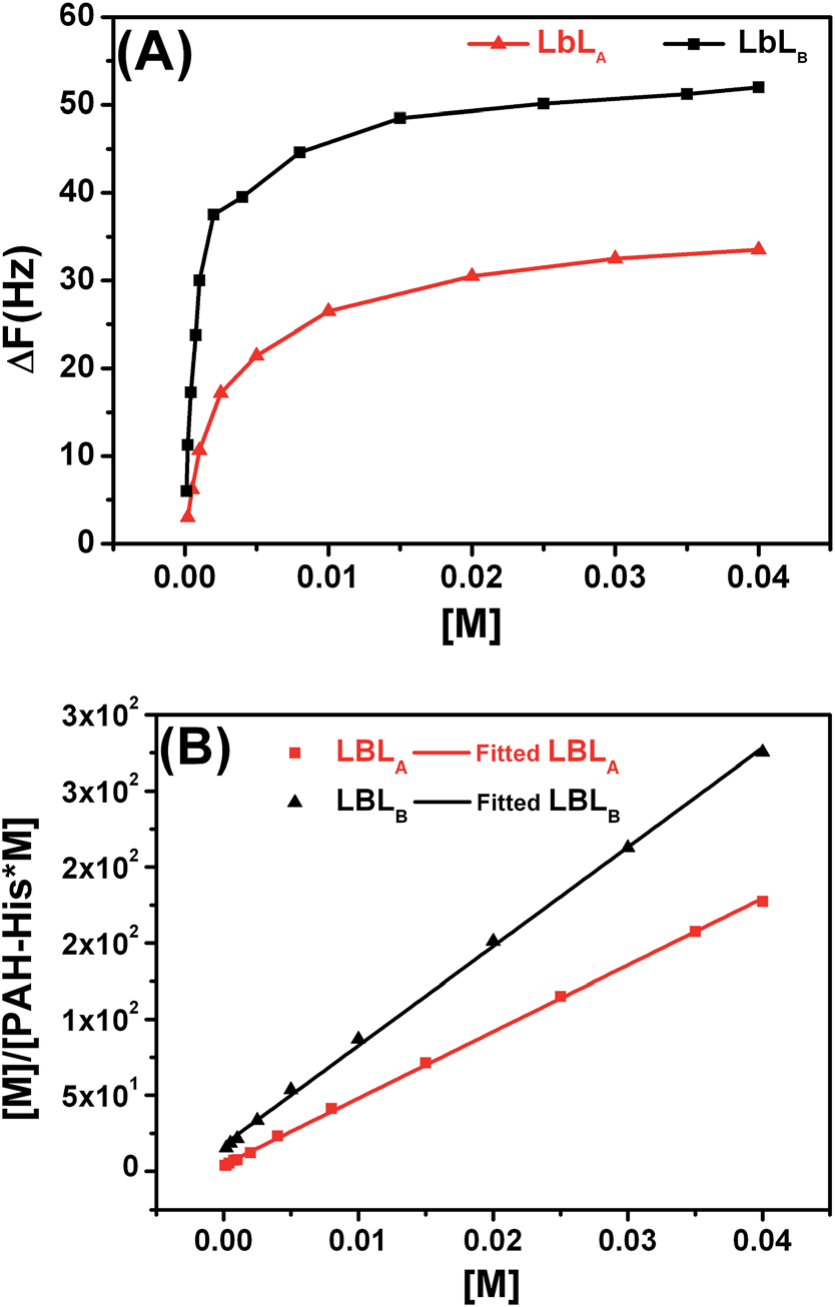
(A) Saturation isotherms monitored by QCM-D for Cu^2+^ loading in non-imprinted LbL_A_ and imprinted LbL_B_ films. The surface proved fully saturable and followed normal Langmuir-like adsorption behavior. Δ*F* represents the frequency change (Hz). (B) Adsorption isotherms of non-imprinted LbL_A_ and imprinted LbL_B_ films for Cu^2+^ ions fitted by the Langmuir model. [M] denotes the molar concentration of Cu^2+^.

Film–metal affinity experiments were carried out by passing Cu^2+^ solutions of different concentrations over the thin film until an equilibrium state was reached (Fig. S7 in the ESI†). The adsorption isotherms obtained in this way display a typical profile of saturation with increased concentration of guest molecules (Fig. 7A). The amount of absorbed mass was calculated from the Sauerbrey equation as stated in the experimental section. The equilibrium adsorbed amount of metal for imprinted LbL_B_ films is 11.25 μg cm^–2^, whereas for non-imprinted LbL_A_ only 4.95 μg cm^–2^ could be immobilized. These observations clearly demonstrate the higher adsorption capacity of the LbL_B_ compared to the non-imprinted LbL_A_ and support the hypothesis of enhanced affinity of the polymeric film to the metal upon imprinting of the receptor structure during the fabrication process.

QCM equilibrium adsorption experiments were conducted to investigate the binding behavior and binding constants for both LbL_A_ and LbL_B_ (Table 1). The saturation isotherms of these films are displayed in Fig. 7A. With the concentration of the copper solution increased, the adsorption capacity of both films first increases sharply then the increase slows down until saturation is reached.

**Table 1:** Equilibrium constants for PAH-His and imprinted and non-imprinted LbL films from different methods

Process	Method	Binding constant	logK
PAH-His (solution)	UV-Visible	*K*_1_ = 8.43 x 10^3^, *K*_2_ = 2.63 x 10^4^	*K*_1_ = 3.93, *K*_2_ = 4.42
Non-imprinted - LbL_A_	QCM (from Langmuir isotherm)	*K*_1_ = 1.04 x 10^3^	*K*_1_ = 3.02
Imprinted - LbL_B_	QCM (from Langmuir isotherm)	*K*_1_ = 7.24 x 10^3^	*K*_1_ = 3.86

A typical method for assessing guest affinity for thin films is based on the application of the simple Langmuir adsorption isotherm model (Fig. 7B). Using this approach we assume a homogeneous distribution of binding sites with equal energy across the film and that the guest diffusion through the thin film is fast and is not the limiting step.^36,41,75^ Learning from initial experiments in solution we also know that both films are interacting with metal ions utilizing predominantly receptors composed of two histidine groups per binding site. In the case of the film affinity this simplifies binding to a 1 : 1 type of interaction but with stoichiometry of two histidine moieties per Cu^2+^. The data obtained in this way (Table 1) displays substantially higher binding affinity of Cu^2+^ for the imprinted LbL_B_ film (logK = 3.9) compared to the non-imprinted LbL_A_ (logK = 3.0).

The Cu^2+^ template-defined organization of PAH-His receptors was protected in the cross-linking process of the LbL_B_ film fabrication. After the template extraction, imprinted LbL_B_ structures remained in place to furnish a spatial configuration of the histidine ligands for the Cu^2+^ coordination. In contrast, enhanced metal binding cavities derived from spatial arrangements of functional groups, and corresponding to Cu^2+^, were absent in the LbL_A_ film due to the lack of metal template in the preparation process. As such LbL_A_ displays randomly immobilized histidine receptors with lower affinity to copper ions compared to LbL_B_.

**Fig. 8:**
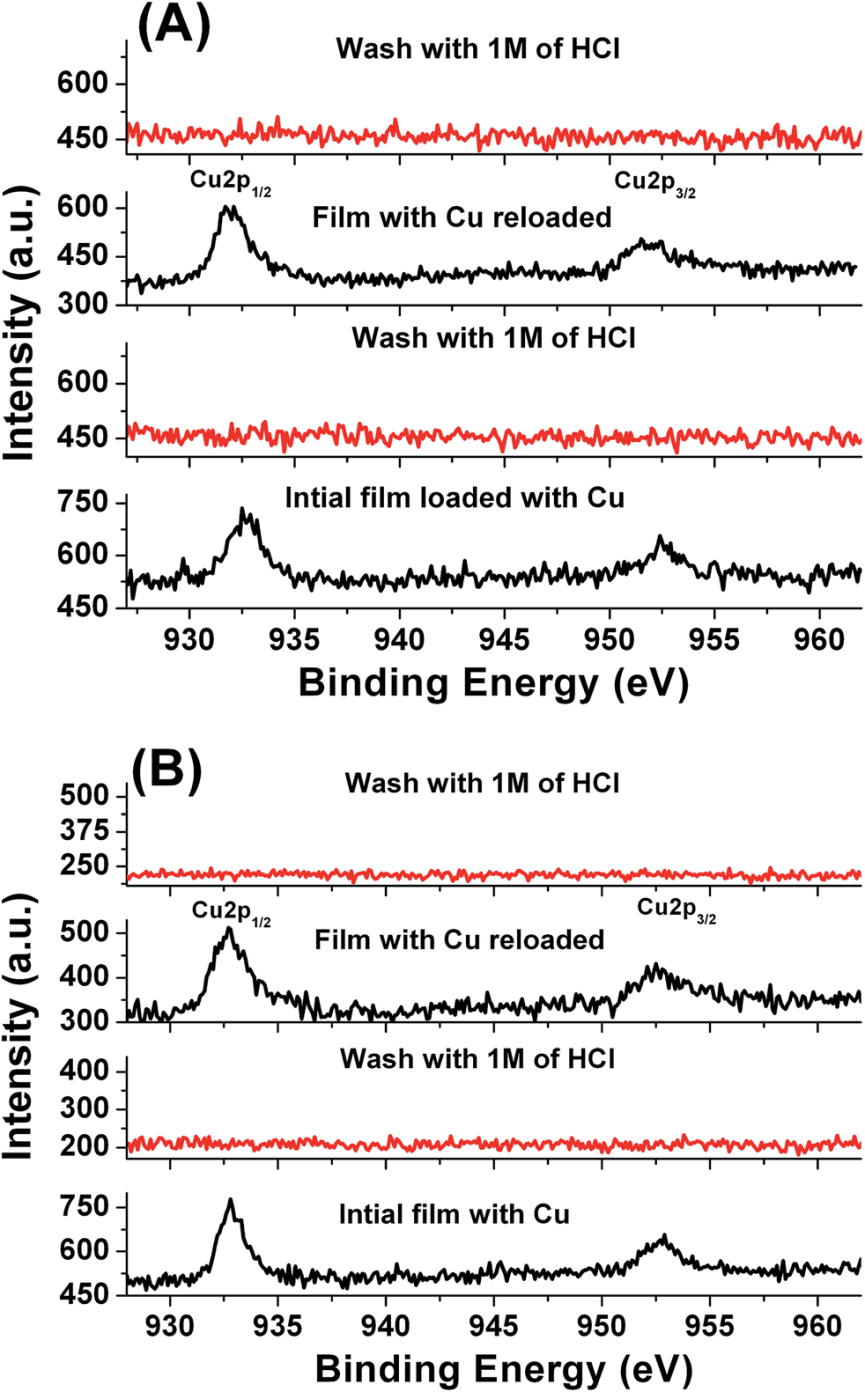
Cu 2p XPS spectra of the LbL film before and after loading of copper in the (A) non-imprinted LbL_A_ films and (B) imprinted LbL_B_ films.

Rebinding capacities of the LbL_A_ and LbL_B_ films were calculated using QCM collected data after washing the films with 1M HCl. Both films showed Cu rebinding levels of 90% and 95% respectively (see the saturation isotherms in the ESI Fig. S8†). A comparison of LbL_A_ and LbL_B_ for affinity toward the Cu^2+^ ion was also investigated using XPS (Fig. 8). The intensities of the Cu2p_3/2_ peak at 933 eV and the Cu2p_1/2_ peak at 953 eV were used for monitoring the trapped copper inside the LbL films.^15^ Copper can be detected for both types of imprinted LbL_A_ and non-imprinted LbL_B_ films, and it can be removed by washing with 1M of HCl for 5 min. The cycle can be repeated several times and these results indicate that both films possess stable affinity toward copper ions.

**Fig. 9:**
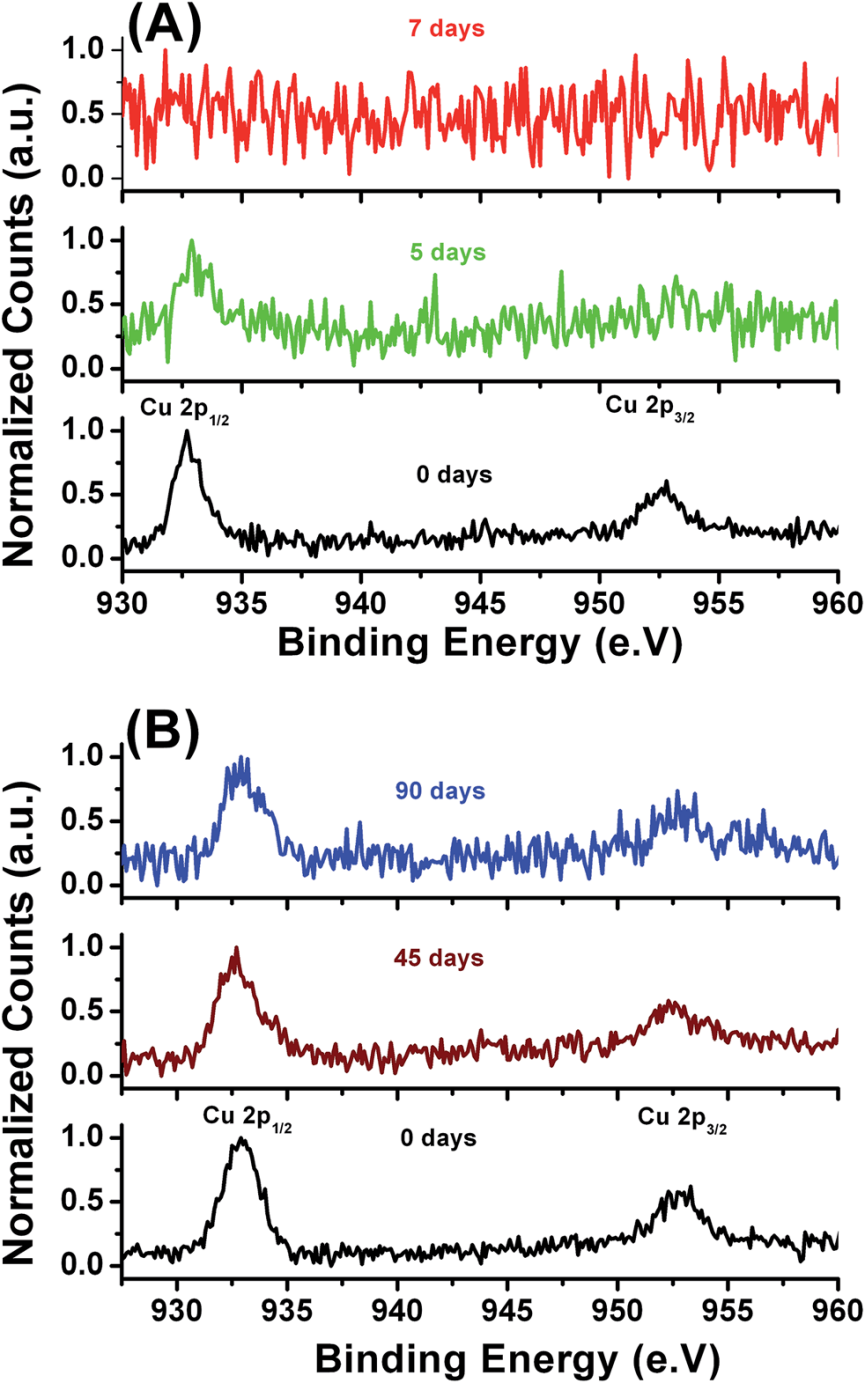
Cu 2p XPS spectra of the non-imprinted LbL_A_ (A) and imprinted LbL_B_ films (B) against sea salts.

The study of the leaching behavior for the metal-bearing films is an important way to obtain valuable information about the chemical properties of the metals in stabilized products and their potential environmental risks in antifouling applications. A better understanding of the leaching rate of the metals trapped inside the LbL films exposed to different environmental conditions is helpful for practical applications. To compare the copper stabilization effect the non-imprinted LbL_A_ and imprinted LbL_B_ films were subjected to a prolonged 90 day leaching experiments (Fig. 9) in an artificial sea water environment.

XPS results provide another confirmation of the enhanced affinity of the imprinted LbL_B_ film toward Cu^2+^ ions. Substantially lower amounts of copper ions leach out of the LbL_B_ film after six weeks of exposure to artificial sea water as compared with the LbL_A_ film. The leaching rate of copper from non-imprinted LbL_A_ is thus obviously faster and all the copper is leached out from the film within a week as shown in the XPS spectra (Fig. 9A). In addition, we have also monitored the N1s XPS spectra of both types of films. The corresponding results (Fig. S9A and B†) showed similar spectra indicating that the films are stable in the sea water environment for a prolonged period of time. Thickness values for both films showed the same values even after 45 days in sea salt solution with minor 5–10% loss after 90 days (measured by ellipsometry). From the above results we can state that the cross-linking between the ester groups from the polyanion and amine groups from the polycation, plays a vital role in improving chemical stability of the films under seawater conditions. Moreover, the presented method can be also used to design surfaces with a customized leaching rate of metal ions in addition to an enhancement of copper binding within the imprinted film.

### Antifouling properties

Barnacles are common, cosmopolitan macrofouling organisms and can rapidly colonize unprotected surfaces submerged in the marine environment. Barnacle larvae (cyprids) are widely used in the lab scale antifouling tests, since cyprids of the barnacles settle readily in static water assays.^76^ In this study a barnacle cyprid bioassay was conducted to evaluate the settlement and toxicity effects of imprinted LbL_A_ and non-imprinted LbL_B_ films with copper.

**Fig. 10:**
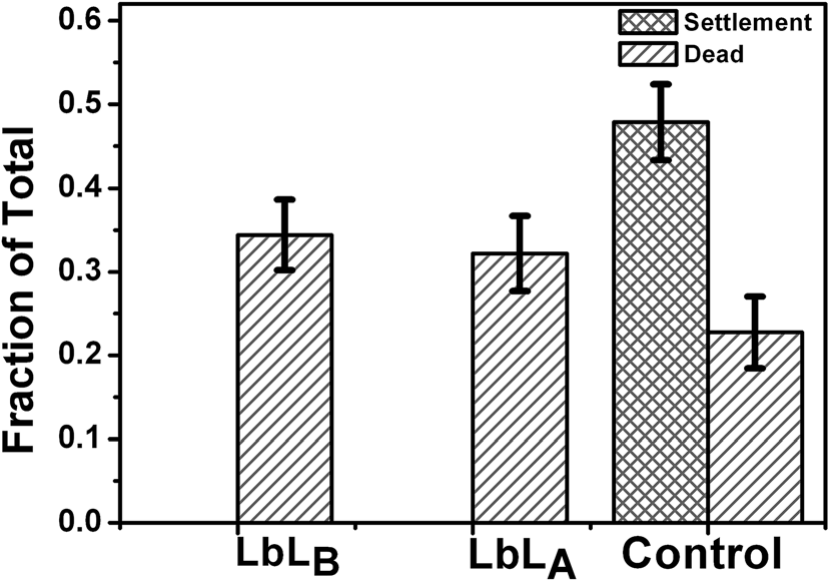
Cyprid settlement and toxicity of imprinted and non-imprinted LbL films with copper loading. Each value is the mean of 8 replicate measurements. The error bars here are standard deviations.

Slightly elevated mortality was observed for naupli incubated for 24 hours on surfaces coated with copper loaded LbL films fabricated with both of the discussed methods compared to the control sample. The lethal concentration (LC_50_) reported for barnacles nauplii is 0.71 μg mL^–1^.^77^ The leached Cu concentration in the model experiment, when the LbL_A_ sample was immersed in 300 μL of water, was established at 60 μg mL^–1^. Thus the amount of copper released from our materials in a typical biological experiment is well below the toxicity threshold. There was no settlement of the cyprids on non-imprinted LbL_A_ and imprinted LbL_B_ films with copper (Fig. 10). These results indicate that Cu^2+^ released from the surface is reaching required fouling preventive concentrations. As the assay is conducted in a static droplet, the concentration of copper present in the seawater droplet is the cumulative value achieved over the 24 hour leaching period.

The statistical comparisons were performed using GraphPad Prism 5 (GraphPad Software Inc.). Cyprid mortality and settlement test data were analyzed using a Kruskal–Wallis test and Dunn's post test to evaluate the performance of the LbL films with copper loading. There is no significant difference between the cyprid mortality on imprinted LbL_A_ and non-imprinted LbL_B_ films and control samples (Kruskal–Wallis test, *p* = 0.1133 and Dunn's post test, *p* > 0.05).^78^ Therefore we can conclude that there is no toxicity difference among those surfaces. However, there was no cyprid settlement for non-imprinted LbL_A_ and imprinted LbL_B_ films. The results suggest that the amount of copper slowly released into solution from the surface was low but sufficient to deter settlement.

## Conclusions

A peptide mimicking polymer, bearing histidine side groups, was used to construct thin LbL architectures with a high affinity to bind Cu^2+^ ions. Enhanced binding ability was achieved through a metal imprinting process involving covalent immobilization of receptors within the film structure in the presence of metal ions. A polycation bearing imidiazole-type histidine ligands for metal binding, and a polyanion with high density of methyl esters for covalent cross-linking were custom synthesized for this task. A comparison of metal imprinted LbL_B_ and non-imprinted LbL_A_ films allowed us to conclude that by following the established approach it is possible to enhance the affinity of thin films to bind copper up to seven-fold. This number is likely to be increased when further process optimization at the fabrication stage is considered. Physical and chemical characterization techniques showed that the prepared imprinted LbL_B_ films display excellent stability under harsh seawater conditions and very slow leaching rate of metals compared to non-imprinted LbL_A_ films.

Finally, the films are promising antifouling materials showing noticeable performance against barnacles. We believe that the presented approach may lead to a generic platform for marine antifouling materials with very low net release rate of biocides. Particularly it may help to reduce the amount of copper currently used in commercial antifouling products. By further improvement of metal ion binding one can imagine that this technology can be directly used to obtain paints with very high metal affinity. For example, absorbing copper in places with a high concentration like harbors or polluted river mouths and then slowly releasing it in an open sea could be envisaged. The fabrication approach presented can also benefit metal extraction processes used for example by the water purification or natural resources recovery industries. The phenomenon described here for copper ions could be extended for other metals like Fe, Zn, Co and Ni and their possible applications could be further explored in heavy metal separations, antifungal layers, protein binding, catalysis and fluorescence sensing.
