# Supplementary material for: Imprinting of metal receptors into multilayer polyelectrolyte films: fabrication and applications in marine antifouling
Source: Chem Sci. 2014 Sep 26;6(1):372–83. doi: 10.1039/c4sc02367f (PMC5586206; doi:10.1039/c4sc02367f)
Supplement: Supplementary file 1 [file SC-006-C4SC02367F-s001.pdf]

Electronic Supplementary Material (ESI) for Chemical Science.
This journal is © The Royal Society of Chemistry 2014

Electronic Supplementary Information:

Imprinting of Metal Receptors into Multilayer Polyelectrolyte Films: Fabrication and Applications in Marine Antifouling

Sreenivasa Reddy Puniredd,[a] Dominik Jańczewski,*[a] Dewi Pitrasari Go, [a] Zhu Xiaoying,[a] Shifeng Guo,[a] Teo Lay Ming Serena,[b] Lee Siew Chen Serina,[b] and G. Julius Vancso, *[cd]

[a] Institute of Materials Research and Engineering, A*STAR (Agency for Science, Technology and Research), 3 Research Link, Singapore 117602

[b] Tropical Marine Science Institute, National University of Singapore, 18 Kent Ridge Road, Singapore 119227

[c] Institute of Chemical and Engineering Sciences, A*STAR, 1, Pesek Road, Jurong Island, Singapore 627833

[d] MESA+ Institute for Nanotechnology, Materials Science and Technology of Polymers, University of Twente, P.O. Box 217, 7500 AE Enschede, the Netherlands

*Tel: +65 6874 5443 (D.J.); +31 53 489 2974 (G.J.V.). Fax: +65 6872 0785 (D.J.); +31 53 4893823 (G.J.V.). E-mail:janczewskid@imre.a-star.edu.sg (D.J.); g.j.vancso@utwente.nl (G.J.V.).

Characterization

The synthesized polymers and all LbL films were characterized by the following methods. The absorption spectra were collected using a UV-VIS spectrometer (UV-3600, Shimadzu). The FTIR measurements were recorded using a Perkin-Elmer FTIR spectroscopy (1600 series II, USA) with an attenuated total reflection component using a ZeSe crystal by accumulating 128 scans at a resolution of 8 cm^{-1}, in the reflectance mode. The sample compartment was purged with nitrogen prior to use. The NMR spectra were collected using a Bruker, 400 MHz. X-ray Photoelectron Spectroscopy (XPS) scans were collected with a VG ESCALAB 250i-XL spectrometer using an Al Kα X-ray source (1486.6 eV photons). The core-level signals were obtained at a photoelectron take-off angle of 90° (with respect to the sample surface). The X-ray source was run at a reduced power of 150 W. The pressure in the analysis chamber was maintained at 7.5×10^{-9} Torr, or lower, during each measurement. Binding energies were calibrated with a carbon reference (C1s, 285 eV). Survey spectra were run in the binding-energy range of 0−1000 eV, and the high-resolution spectra of C1s, N1s, and Cu 2p were collected. The surface morphology of the films was examined by Atomic Force Microscopy (AFM) (NanoWizard II from JPK Instruments AG, Berlin, Germany) equipped with a NanoWizard head and controller. All images were collected in air using the tapping mode under ambient

conditions and standard silicon probes (k ~ 40 N/m, Tap 300AL-G, Budget sensors). AFM images were taken on dried films with an area of 5 μm × 5 μm for morphology observations and roughness measurements. The film thickness was measured by scratching the multilayer assembly with a fresh razor blade to expose the bare substrate (silicon) and then scanning it over an area of 10 μm × 10 μm to reveal a clear step at the scratch.[1] The height difference between the thin film surface and the bare substrate was considered as the thickness of the thin film. Three to five sections crossing of a single scratch were used to calculate mean value of the height differences used as the film thickness. AFM raw data was processed by the software NanoScope Analysis, Bruker.[1] Thickness characterization was also performed by spectroscopic ellipsometry (Wvase 32, Woollam). Scanning spectra were acquired over the wavelength range of 600-1000 nm at incidence angles of 65°, 70° and 75°. For the modeling of the ellipsometric measurements silicon (Si.MAT) a base layer with 0.6 mm thickness was used, followed by a silicon dioxide (SiO2.MAT) as the next layer with a thickness of 2.5 to 3 nm. The Cauchy package (CAUCHY.MAT) was utilized for data fits. The ellipsometric thicknesses of the LbL films were determined by averaging five spots on each sample. The mean-squared error (MSE) values were less than 2 nm after fitting, indicating reliability of the model and the measurement.

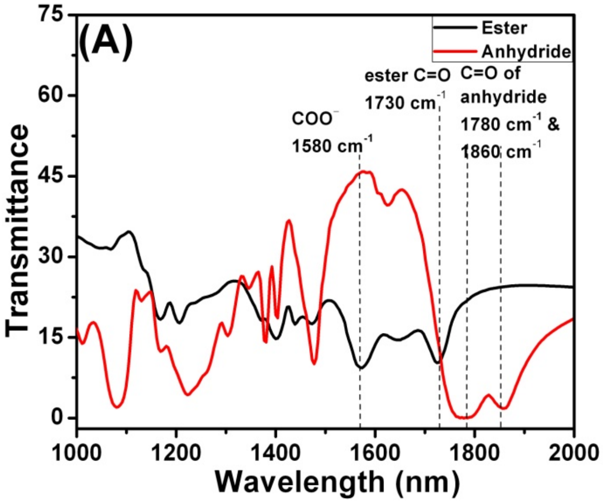

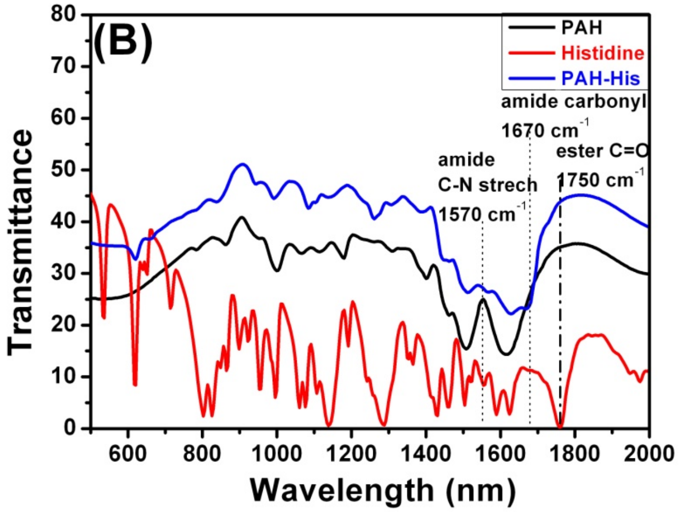

Figure S1. FTIR spectra of pure anhydride and after ester conversion (Figure S1A), and pure PAH, Histidne and PAH-His(Figure S1B).

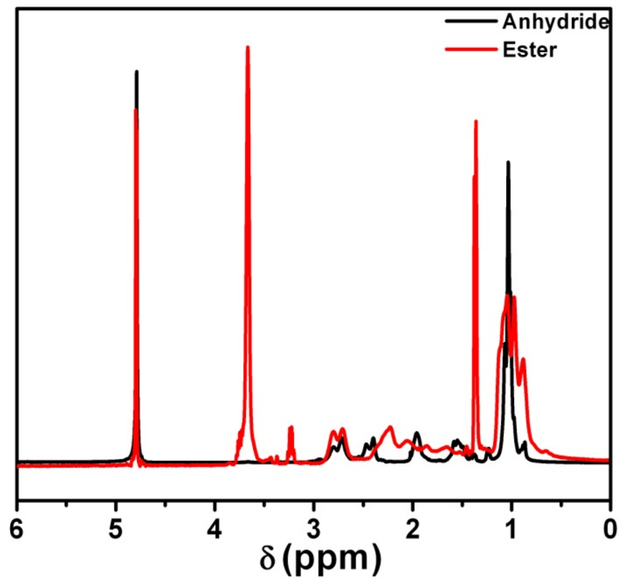

Figure S2. ^{1}H NMR spectra of anhydride (black) and after conversion of ester (PIAMA-Me, red).

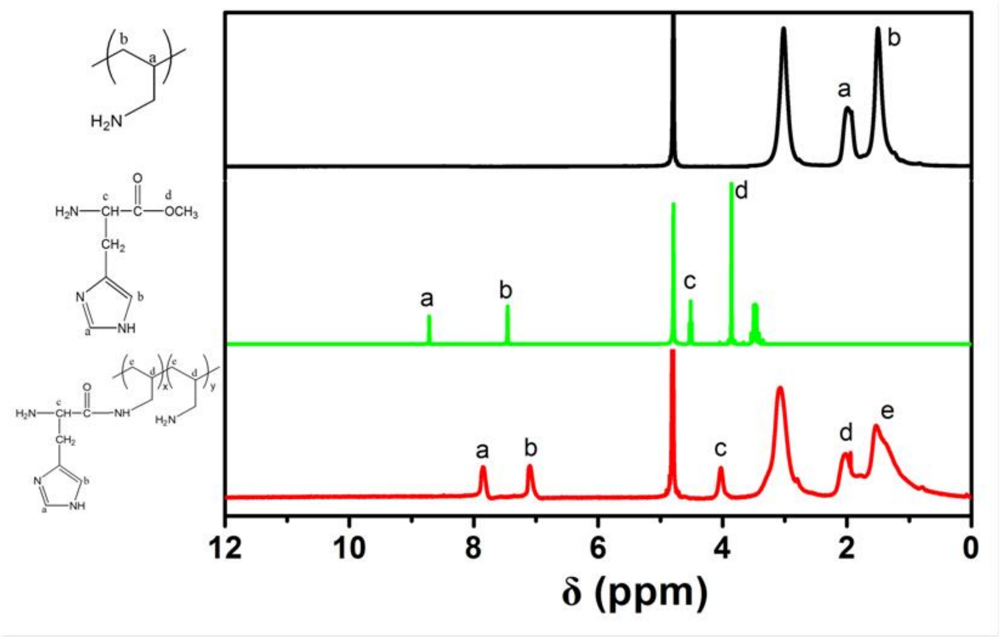

Figure S3. ^{1}H-NMR spectra of PAH (black line), Histidine (green line), and PAH-His (red line, 20% of Histidine substitution degree) in D_2O.

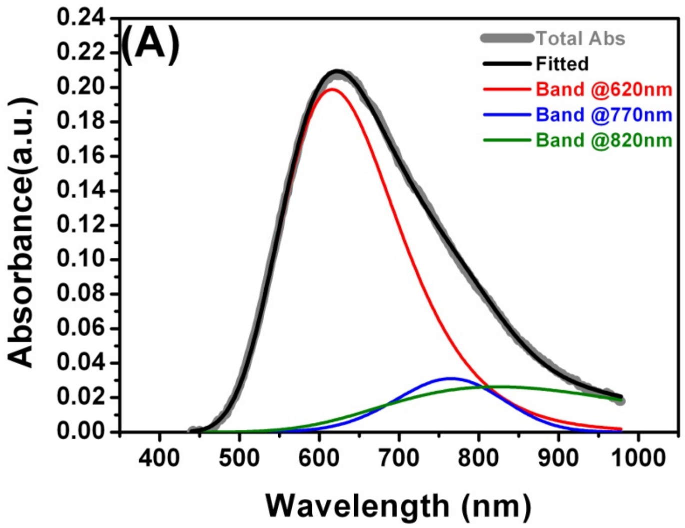

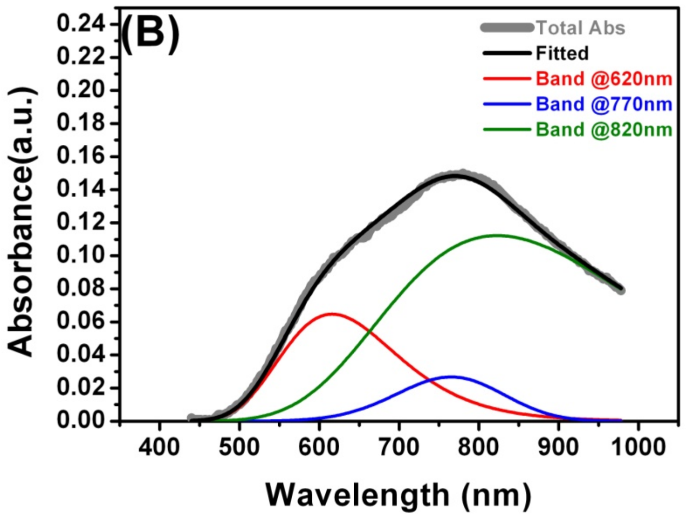

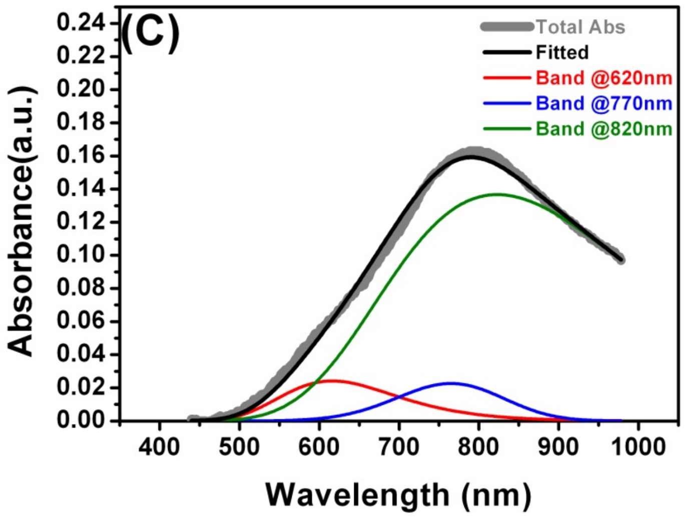

Figure S4. Representative UV-Visible absorption spectra of three curves fitted at 620nm, 770nm and 820 nm for (A)0.6 ml (B) 4.5 ml and (C)9.8 ml. of PAH-His(6 mM) added to the 2ml of $Cu(NO_3)_2$(15 mM)

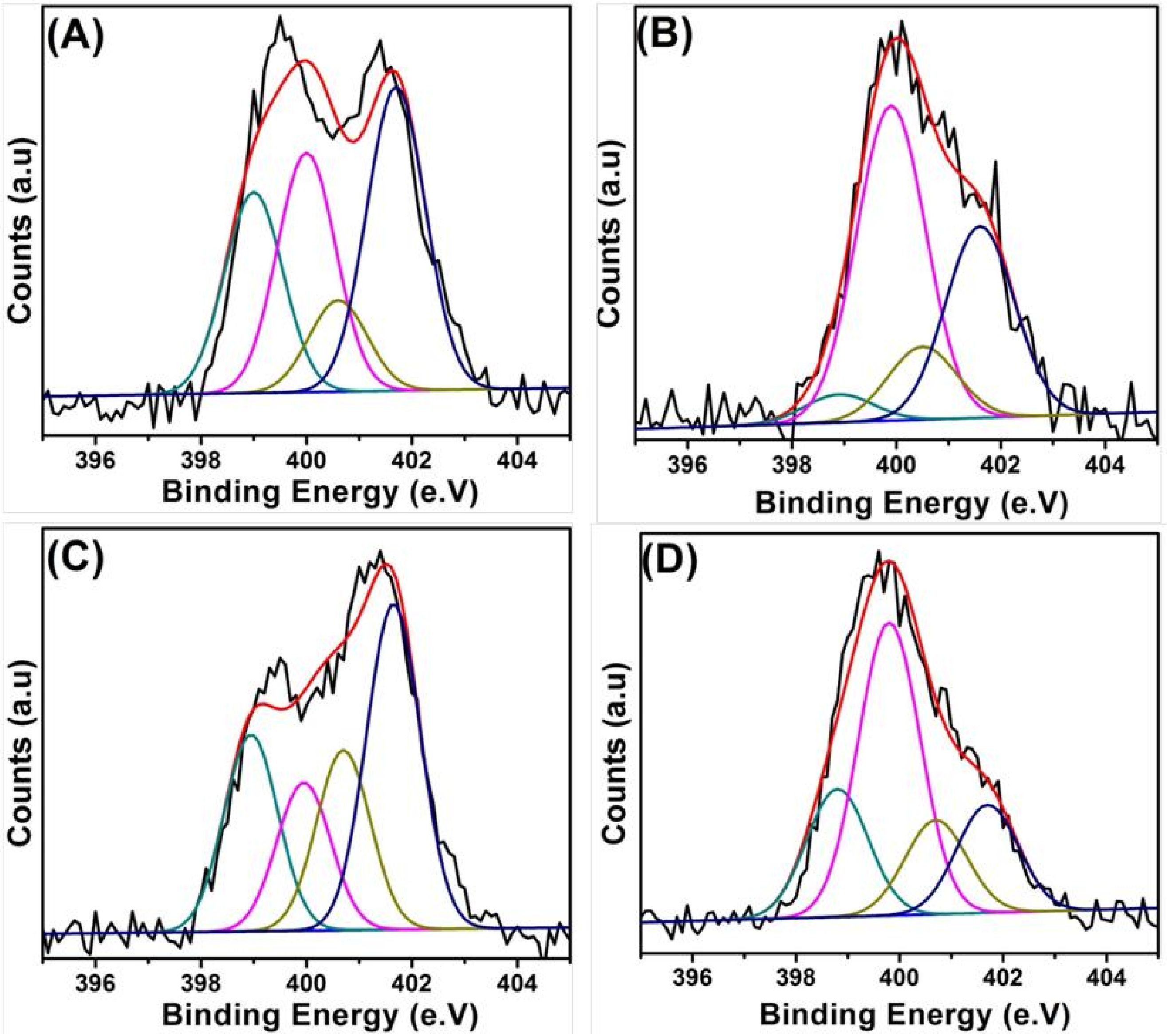

Figure S5. XPS spectra of the non cross linked (A and C for non-imprinted LbL_A and imprinted LbL_B) and cross-linked (B and D for non-imprinted LbL_A and imprinted LbL_B) films respectively.

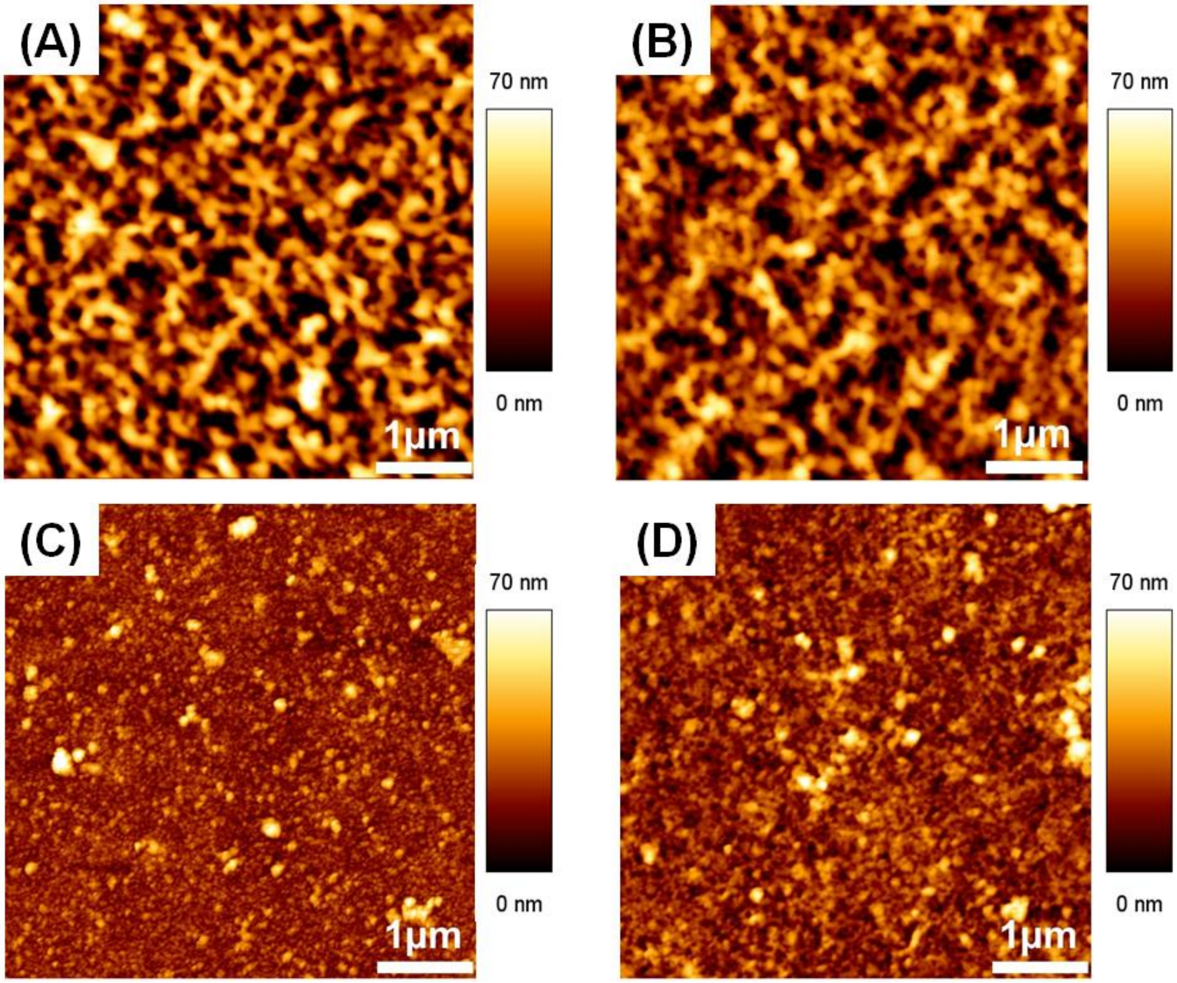

Figure S6. Tapping mode AFM phase images of the non-imprinted LbL_A films of 7 bi-layers ((A) before cross linking, (B) after cross linking) and imprinted LbL_B of 10 bi-layers film ((C) before cross linking, (D) after cross linking).

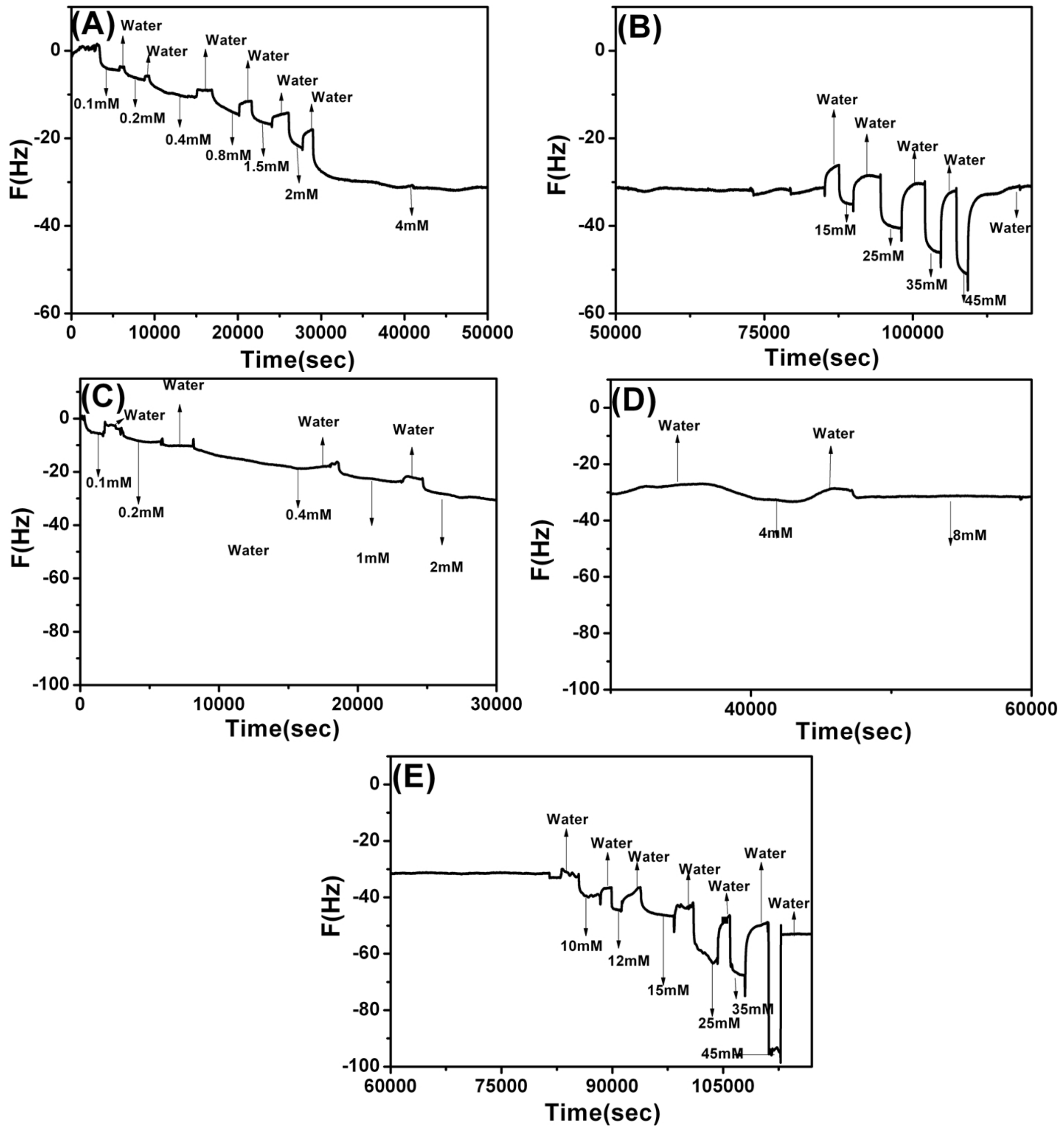

Figure S7. Magnified frequency changes monitored by QCM-D for non-imprinted LbL_A (A and B) and imprinted LbL_B (C, D and E) films with different concentration of $Cu(NO_3)_2$ loading.

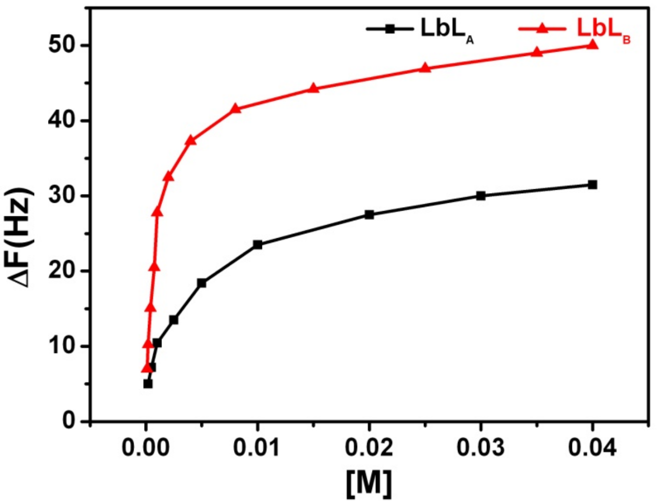

Figure S8. Second cycle of saturation isotherm monitored by QCM-D for copper loading in non-imprinted LbL_A and imprinted LbL_B films .

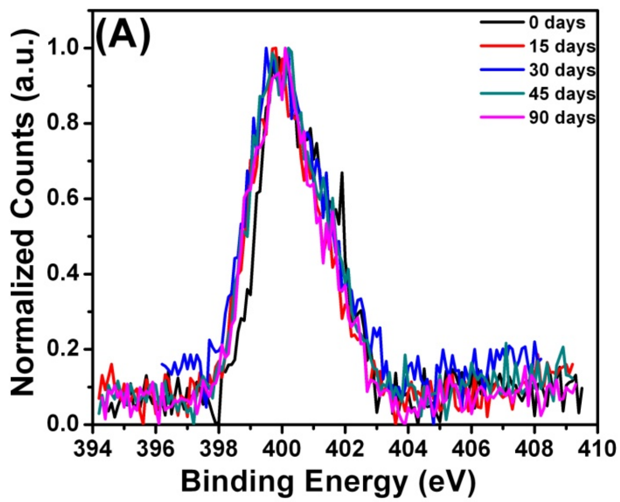

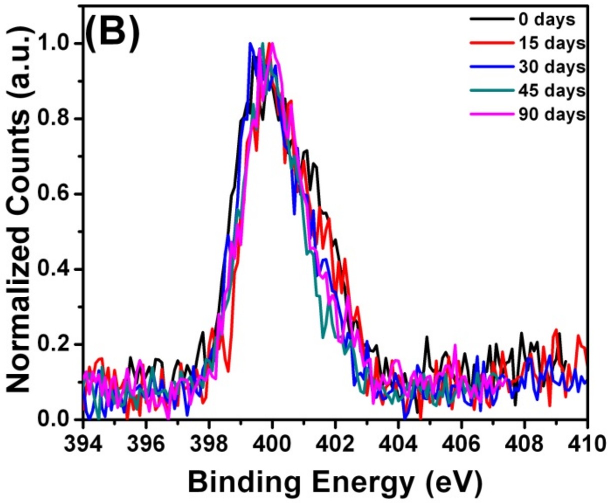

Figure S9. N1s, XPS spectra of the LbL films processed my non-imprinted LbL_A (Figure S9A) and imprinted LbL_B films (Figure S9B) against sea salts.

1. X. Zhu, D. Jańczewski, S. S. C. Lee, S. L.-M. Teo and G. J. Vancso, *ACS Appl. Mater. Interfaces*, 2013, 5, 5961−5968.
